# Clade-age-dependent diversification under high species turnover shapes species richness disparities among tropical rainforest lineages of *Bulbophyllum* (Orchidaceae)

**DOI:** 10.1186/s12862-019-1416-1

**Published:** 2019-04-24

**Authors:** Alexander Gamisch, Hans Peter Comes

**Affiliations:** Department of Biosciences, University of Salzburg, Hellbrunnerstrasse 34, 5020 Salzburg, Austria

**Keywords:** Diversification, Ecological niche modelling, Orchidaceae, Species richness disparity, Tropical rainforest, Turnover

## Abstract

**Background:**

Tropical rainforests (TRFs) harbour almost half of the world’s vascular plant species diversity while covering only about 6–7% of land. However, why species richness varies amongst the Earth’s major TRF regions remains poorly understood. Here we investigate the evolutionary processes shaping continental species richness disparities of the pantropical, epiphytic and mostly TRF-dwelling orchid mega-genus *Bulbophyllum* (*c.* 1948 spp. in total) using diversification analyses based on a time-calibrated molecular phylogeny (including *c.* 45–50% spp. each from Madagascar, Africa, Neotropics, and 8.4% from the Asia-Pacific region), coupled with ecological niche modelling (ENM) of geographic distributions under present and past (Last Glacial Maximum; LGM) conditions.

**Results:**

Our results suggest an early-to-late Miocene scenario of ‘out-of-Asia-Pacific’ origin and progressive, dispersal-mediated diversification in Madagascar, Africa and the Neotropics, respectively. Species richness disparities amongst these four TRF lineages are best explained by a time-for-speciation (i.e. clade age) effect rather than differences in net diversification or diversity-dependent diversification due to present or past spatial-bioclimatic limits. For each well-sampled lineage (Madagascar, Africa, Neotropics), we inferred high rates of speciation and extinction over time (i.e. high species turnover), yet with the origin of most extant species falling into the Quaternary. In contrast to predictions of classical ‘glacial refuge’ theories, all four lineages experienced dramatic range expansions during the LGM.

**Conclusions:**

As the Madagascan, African and Neotropical lineages display constant-rate evolution since their origin (early-to-mid-Miocene), Quaternary environmental change might be a less important cause of their high species turnover than intrinsic features generally conferring rapid population turnover in tropical orchids (e.g., epiphytism, specialization on pollinators and mycorrhizal fungi, wind dispersal). Nonetheless, climate-induced range fluctuations during the Quaternary could still have played an influential role in the origination and extinction of *Bulbophyllum* species in those three, if not in all four TRF regions.

**Electronic supplementary material:**

The online version of this article (10.1186/s12862-019-1416-1) contains supplementary material, which is available to authorized users.

## Background

**Fig. 1:**
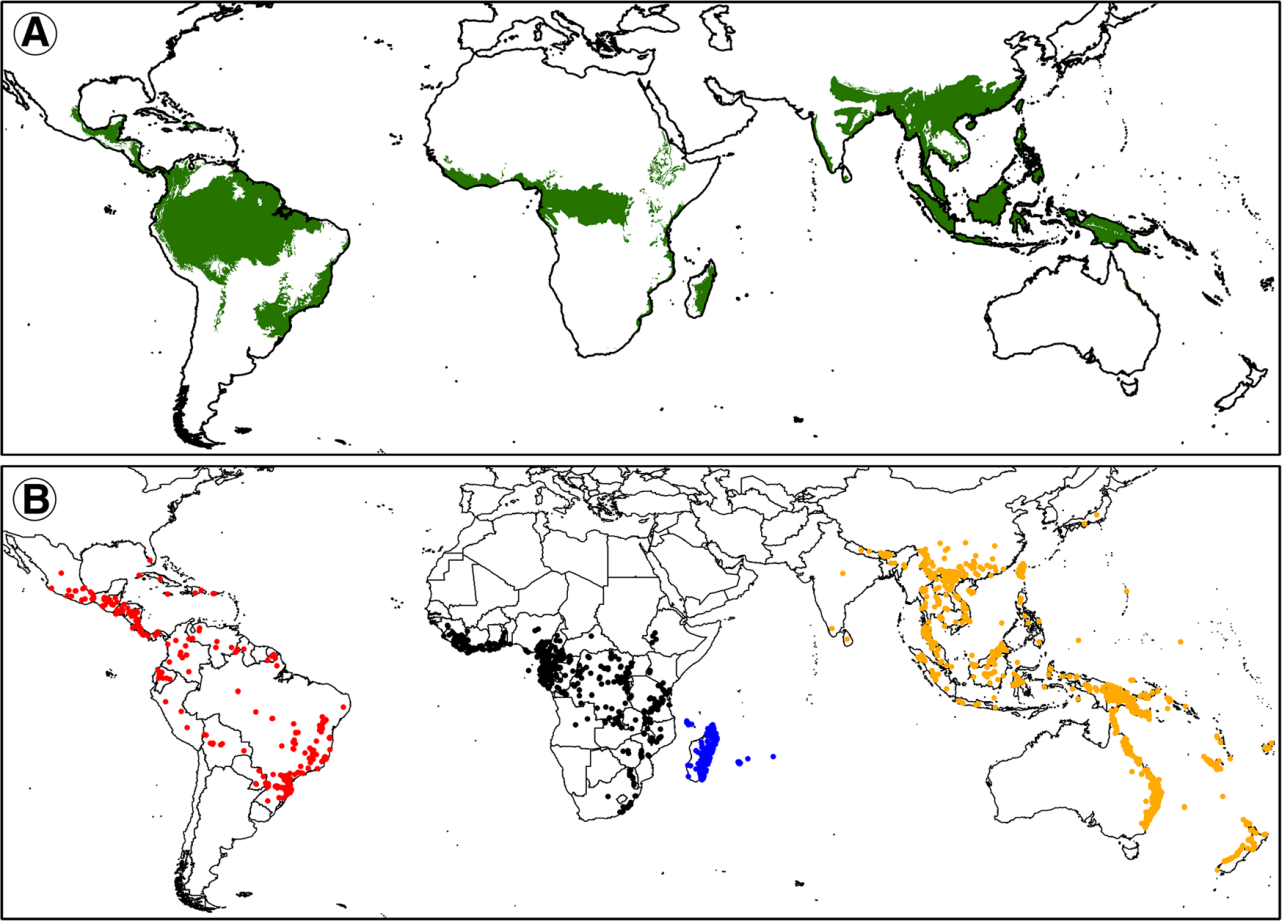
Distribution of the pantropical orchid genus *Bulbophyllum* across the four major tropical rainforest (TRF) regions. **a** Distribution of tropical rainforests sensu *lato* (tropical and subtropical moist broadleaf forest) based on Olsen and Dinerstein [3] as freely available from The Nature Conservancy website (http://maps.tnc.org). **b** Extant occurrence points (dots) of the genus in the Neotropics (red), Africa (black), Madagascar (blue) and the Asia-Pacific region (orange), based on GBIF geo-referenced specimens and additional records taken from the literature and herbarium collections (see text). The maps were generated using ArcGIS v. 10.4.1

Despite covering only about c. 6-7% of the Earth’s land surface (*c.* 8.3 × 10^8^ ha in total), tropical rainforests (TRFs) are by far the most species-rich terrestrial ecosystems, with about 175,200 species of vascular plants [1, 2]. The majority of TRFs are found in three biogeographic regions [3, 4]: the Neotropics (e.g., Amazonian Basin, Atlantic Forest), mainland Africa (e.g., Congo River Basin), and the Asia-Pacific region (Asia, New Guinea, Australia, Pacific Islands; Fig. 1a). However, both species diversity (richness) and TRF area are not evenly distributed among those regions. Rather, it is estimated [4] that the African forests are somewhat smaller and far less diverse (1.8 × 10^8^ ha; *c.* 16,000 spp.) than those in the Asia-Pacific region (2.5 × 10^8^ ha; *c.* 61,700 spp.), while none of those estimates rivals the extraordinarily high richness of the vast Neotropical forests (4.0 × 10^8^ ha; *c.* 93,500 spp.). Remarkably though, despite covering a much smaller area (*c*. 4.2–8.8 × 10^6^ ha [5]), the TRF of the island of Madagascar is surprisingly species-rich (*c.* 7600 spp. [6]). Hence, Madagascar is often considered a TRF region on its own [7] (Fig. 1a).

Together, these four TRF regions play an invaluable role in sustaining high levels of global biodiversity [8] while being increasingly threatened by various types of human disturbance and climate change [9]. Hence, explaining the evolutionary and ecological causes of TRF richness patterns between the highly diverse and disjunct regions of the Neotropics, Africa, Madagascar and the Asia-Pacific region is particularly important for the understanding of modern biodiversity and its conservation. Yet, why some of those regions have higher or lower diversity remains an unresolved question [10, 11], even though numerous explanations have been advanced.

For example, the markedly lower diversity of tropical Africa (the ‘odd man out’ pattern sensu Richards [12]) is commonly thought to reflect more severe TRF range contractions viz. higher extinction rates during (Late) Quaternary cool/dry periods [11, 13, 14], such as experienced during the Last Glacial Maximum (LGM; *c*. 21,000 years ago) with *c.* 84% of the TRF area reduced in comparison to the American tropics (54%) [15] and the Asian tropics (*c.* 66% [16]; but see Cannon et al. [17]). Similarly, for Madagascar, high levels of rainfall, associated with the island’s eastern mountain ranges, might have buffered TRF-dwelling species from extinction during such drying periods [4, 18]. Moreover, a recent phylogenetic study suggested that the outstanding diversity of angiosperms in the Neotropics might result from ‘rapid evolutionary turnover’ viz. high speciation and extinction rates [19]. At the very least, this might reflect more recent and rapid radiations, for instance driven by Andean uplift [11, 20] or climate-induced range fragmentation [21, 22], in comparison with the African and Asian tropics [10] (reviewed in Richardson and Pennington [11]). Finally, it has also been shown that phylogenetic diversity within a given tropical region not always results from in situ diversification but can also be increased (viz. confounded) by dispersal from elsewhere [23] (reviewed in Eiserhardt et al. [2]). However, there is a general paucity of formal comparative studies between the Neotropics, mainland Africa and the Asia-Pacific region [2, 10], and no taxon-based phylogenetic study to date has explicitly included Madagascar in these global perspectives of tropical richness patterns and diversification processes.

Any interpretation of regional variation in species richness patterns is confronted with the challenge to unravel a complex set of potentially underlying and interacting factors, including phylogenetic/biogeographic history, current and past environmental (geological, geographical, climatic, etc.) conditions, or the origin of trait novelties [24]. On the other hand, disentangling the causes of such variation has seen a large body of large-scale phylogenetic comparative research, especially with focus on the negative latitudinal (tropical vs. temperate) biodiversity gradient [25–29] or amongst temperate (e.g., Mediterranean-type climate) regions [30, 31]. Based on those and similar studies [32], three mutually non-exclusive processes have been proposed that should be ultimately responsible for a clade’s higher diversity in a given area: (1) higher rates of net diversification; (2) a longer time period available to accumulate species (the clade age or time-for-speciation effect [25]), including the time of in situ diversification or the time since colonization of a region [28, 33]; and/or (3) a higher spatial-ecological limit (or ‘carrying capacity’) to diversification [34, 35]. Under this latter hypothesis, constraints imposed by geographical area and/or niche availability may eventually slow down the diversification process (via increased extinction and/or reduced speciation) as a lineage accumulates species over time (reviewed in Moen and Morlon [36]).

Although phylogenetic studies statistically testing the diversification of pantropically distributed TRF plant taxa are scarce [2], several have documented diversification rates (e.g., Arecaceae [37]; Annonaceae [38]; *Manilkara* (Sapotaceae) [22]; Protieae (Burseraceae) [39]; angiosperms [19]; Orchidaceae [40]). Some of these studies also compared rates of diversification between certain continental-tropical regions (i.e. Neotropics, Africa, Asia [22]; Neo- vs. Palaeotropics [19]; Neotropics, Africa, Southeast Asia, Australia, Pacific [40]); however, they neither considered the potential role of a time-for-speciation effect between regions nor addressed how geographical area itself, or a proxy measure of niche availability (e.g., climatically suitable area predicted by ecological niche models (ENMs) [35]), relate to species richness and/or diversification rate. In fact, we are unaware of any study that has examined the roles of all three processes potentially driving plant species richness disparities amongst tropical regions in general, and the four main TRF regions in particular, i.e. speciation/extinction, time-for-speciation and spatial-ecological limits.

Here, we address these issues in the pantropical, mostly epiphytic orchid genus *Bulbophyllum* Thouars (Epidendroideae, Dendrobieae; Fig. 1b). This is one of the largest genera of flowering plants, comprising *c.* 1948 species [41], which are predominantly restricted to rainforest habitats [42]. As typical for epiphytes, greatest abundance of individuals and species diversity of *Bulbophyllum* occurs at mid-elevations, where fog and clouds provide ideal growing conditions [43]. However, species diversity varies markedly between the four major TRF regions [41]: it is extremely high in the Asia-Pacific region (*c.* 80.3% of species), while Madagascar still harbours higher diversity (*c.* 10.8%) in comparison to both the Neotropics (*c.* 4.8%) and mainland Africa (*c.* 4.1%). This uneven spread and relatively high number of species in Madagascar makes *Bulbophyllum* a particular interesting candidate for testing hypotheses about richness disparities amongst TRF regions.

To date, most species-level molecular phylogenies for *Bulbophyllum* have focussed on particular regions and smaller sub-generic groups (Asia [44–46]; Madagascar [47–49]; Neotropics [50]). Recently, however, Gamisch et al. [48] derived a time-calibrated phylogeny for the genus based on DNA sequences from the internal transcribed spacer (ITS) regions of nuclear ribosomal (nr) DNA to estimate the crown age of a particular Madagascan lineage (‘clade C’; *c.* 30 spp.). This tree, containing about 13.4% of the genus’ total species diversity (*c.* 262 out of 1948 spp. [41]), with particularly comprehensive samplings of Madagascar, Africa and the Neotropics (*c.* 56.1% of the total across these regions; 220/392 spp.), indicated that *Bulbophyllum* (1) consists of four major clades that are geographically largely coherent within each TRF region as consistent with morphological evidence (see also Pridgeon et al. [42]); (2) has a stem age of *c*. 29.3 million years ago, Ma (95% highest posterior density, HPD: 23.3–37.0 Ma; see also the dated orchid genus-level phylogeny of Givnish et al. [40, 51]); (3) originated in the Asia-Pacific region; and (4) expanded from there to Madagascar, Africa and the Neotropics (see also Givnish et al. [40]), even though the sequence and mode of biogeographic events remain unclear.

Here, we enlarge and further analyse the ITS dataset of Gamisch et al. [48] to (1) infer the genus’ large-scale historical biogeography in more detail; (2) compare rates of diversification amongst the TRF regions using information on branching times [52] (only for the Madagascan, African and Neotropical lineages) as well as clade size and age [53] (all four lineages); and (3) test for the influence of both clade ages and potentially suitable area viz. ENM-derived climatic niche (for the present and the LGM) on these rates as well as patterns of regional species richness. These analyses allowed us to explore the evolution of TRF biodiversity by testing which of the three major processes outlined above (diversification, time-for-speciation, spatial-ecological viz. -bioclimatic limits) had the strongest effect on among-region variation in species richness in this pantropical orchid genus. In addition, our region-specific ENMs for *Bulbophyllum* at the present and the LGM provide novel insights into how climate-induced range dynamics during the Quaternary may have influenced regional diversification patterns and modes of speciation/extinction in tropical biota, a topic that still remains controversial (e.g., [14, 54]).

## Results

### Phylogeny reconstruction and divergence time estimates

**Fig. 2:**
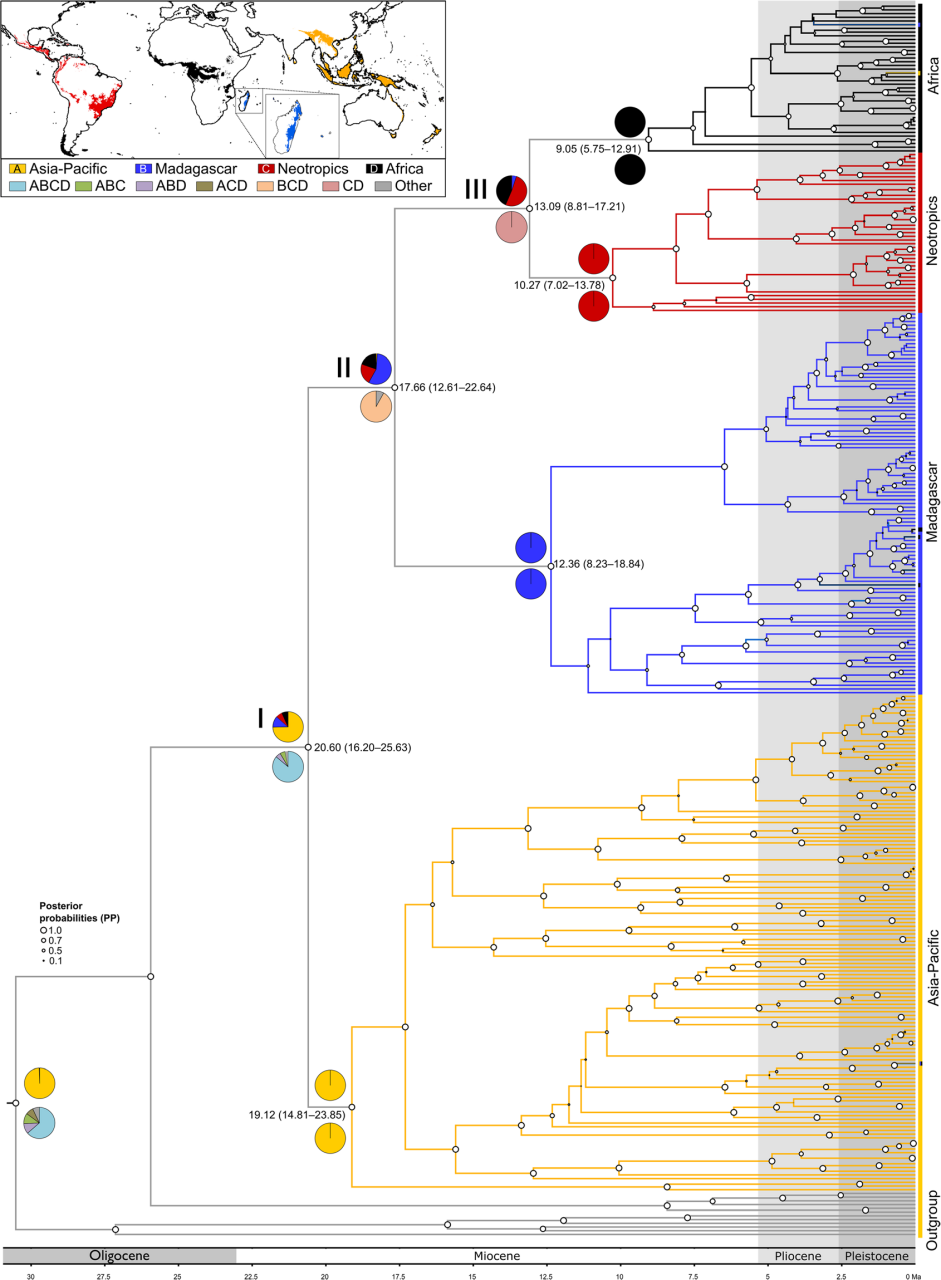
Chronogram and biogeographic reconstructions of *Bulbophyllum*. BEAST-derived species-level maximum clade credibility (MCC) chronogram of *Bulbophyllum* (plus outgroups) based on ITS sequence data, with branch lengths proportional to time reflecting the established relationships of the four regional lineages (see also Pridgeon et al. [42]). Median node ages (in millions of years ago, Ma) and their 95% highest posterior density (HPD) intervals are indicated at nodes of interest (see also Additional file 1: Figure S1). White circles at nodes indicate Bayesian posterior probabilities (PP) [see also Additional file 1: Figure S2 for all numerical PP values]. Pie charts above and below nodes of interest show the relative probabilities of each possible range configuration as obtained from BAYESTRAITS and BIOGEOBEARS (DEC model), respectively. The insert map shows the geographical distribution of the four regional lineages (colour coded), with the key identifying extant and possible ancestral ranges. Note that each terminal branch represents a single extant species

In line with earlier phylogenetic estimates [42, 48], our BEAST-derived phylogeny of *Bulbophyllum*, based on ITS sequence data (Fig. 2), recovered the Asia-Pacific and Madagascan lineages as successive sister groups to a clade comprised of the African and Neotropical lineages (posterior probabilities, PP = 0.97–1.00), with all internal relationships relatively well resolved (i.e. *c*. 59% of internal nodes received PP values of 0.90–1.00; see Fig. 2, Additional file 1: Figure S2). Based on our molecular dating (Fig. 2, Table 1), median posterior estimates of stem and crown ages of these four major lineages were centred on the Early to Late Miocene, *c.* 21–9 Ma. More specifically, these results suggested that: (1) the temporal origin and radiation of the Asia-Pacific lineage occurred in quick succession during the Early Miocene, *c.* 20.60 (95% HPD: 16.20–25.63) Ma and 19.12 (14.81–23.85) Ma, respectively; (2) the younger, mid-Miocene radiation of the Madagascan lineage, *c.* 12.36 (8.23–18.84) Ma, coincided with the divergence of the Neotropical and African lineages, *c.* 13.09 (8.81–17.21) Ma; and (3) both latter clades started to diversify almost synchronously at the beginning of the Late Miocene, *c.* 10.27 Ma and 9.05 Ma (7.02–13.78 and 5.75–12.91 Ma), respectively. However, the great majority of extant species of the non-Asian lineages (154/186, *c.* 82.8%) likely originated during the Quaternary (≤ 2.6 Ma; Fig. 2), and the same applies to those species representing the less well-sampled Asia-Pacific lineage (84/132, *c.* 63.6%).

**Table 1:** Stem and crown ages (in million of years ago, Ma) and diversification rate estimates of the four regional *Bulbophyllum* lineages and their potentially suitable areas under current climatic conditions (*c.* 1950–2000; see Fig. 6a) and those of the Last Glacial Maximum (LGM; *c.* 21,000 years ago), as inferred from two climate models, CCSM and MIROC, and their consensus projection (see Fig. 6b)

Clade	*N*	Stem age	Crown age	Area				BAYESRATE			Magallón and Sanderson [53]			
		(95% HPD)	(95% HPD)	current	LGM	LGM	LGM	*r* (95% HPD)	*μ* (95% HPD)	*λ* (95% HPD)	*r* (*e* = 0)	*r* (*e* = 0)	*r* (*e* = 0.9)	*r* (*e* = 0.9)
					CCSM	MIROC	Consensus				Stem	Crown	Stem	Crown
Madagascar	210	17.66 (12.61–22.64)	12.36 (8.23–18.84)	7032	11,478	16,112	14,470	0.34 (0.20–0.50)	0.72 (0.29–1.21)	1.06 (0.69–1.44)	0.30	0.38	0.17	0.25
Africa	80	13.09 (8.81–17.21)	9.05 (5.75–12.91)	127,851	180,560	256,878	226,176	0.41 (0.22–0.59)	0.29 (0.0013–0.72)	0.70 (0.42–1.06)	0.33	0.41	0.17	0.24
Neotropics	94	13.09 (8.81–17.21)	10.27 (7.02–13.78)	140,456	349,980	468,849	397,909	0.27 (0.08–0.45)	0.80 (0.17–1.5)	1.08 (0.56–1.73)	0.34	0.37	0.18	0.22
Asia-Pacific region	1564	20.60 (16.20–25.63)	19.12 (14.81–23.85)	237,204	305,970	356,731	344,817	NA	NA	NA	0.36	0.35	0.25	0.26

Abbreviations: *N*, number of extant species (according to Sieder et al. [41]); *λ*, speciation rate; *μ*, extinction rate; *r*, net diversification rate (*λ–μ*); *ε*, extinction fraction (*μ*/*λ*); HPD, highest posterior probability

Area was estimated as the number of grid cells above the mean maximum training sensitivity plus specificity (MTSS) logistic threshold. Diversification rates were estimated using information on branching times (BAYESRATE) or clade age (either stem or crown) and clade size [53]

### Ancestral area reconstructions

Ancestral area reconstructions in BAYESTRAITS (Fig. 2) identified the Asia-Pacific region (‘A’) as the genus’ ancestral area (crown node I in Fig. 2) with relative high probability (PP = 75) and ‘decisive’ evidence (BF = 11.28–15.63; see Additional file 1: Table S2). In addition, Madagascar (‘B’) was reconstructed as the most probable state for the stem node (II) of the Madagascan+African+Neotropical lineage (PP = 57), albeit with only ‘weak’ evidence (BF = 1.27–1.78). Finally, both the Neotropics (‘C’) and Africa (‘D’) were assigned with near equal probability to the stem node (III) of these sister lineages (PP = 51 and 44, respectively), again, resulting in ‘weak’ evidence for this node (BF = 0.20; Additional file 1: Table S2). By contrast, the best-fitting DEC model in BIOGEOBEARS (Fig. 2) consistently inferred combined areas at nodes I–III with high relative probability (i.e. node I: ‘ABCD’, 0.95; node II: ‘BCD’, 0.92; node III: ‘CD’, 1.0). In sum, BAYESTRAITS suggested that *Bulbophyllum* arrived in Madagascar from the Asia-Pacific region, and further dispersed to Africa and the Neotropics (or vice versa), whereas the DEC model inferred a once widespread ancestor subject to a series of vicariant events.

### Diversification analyses

**Fig. 3:**
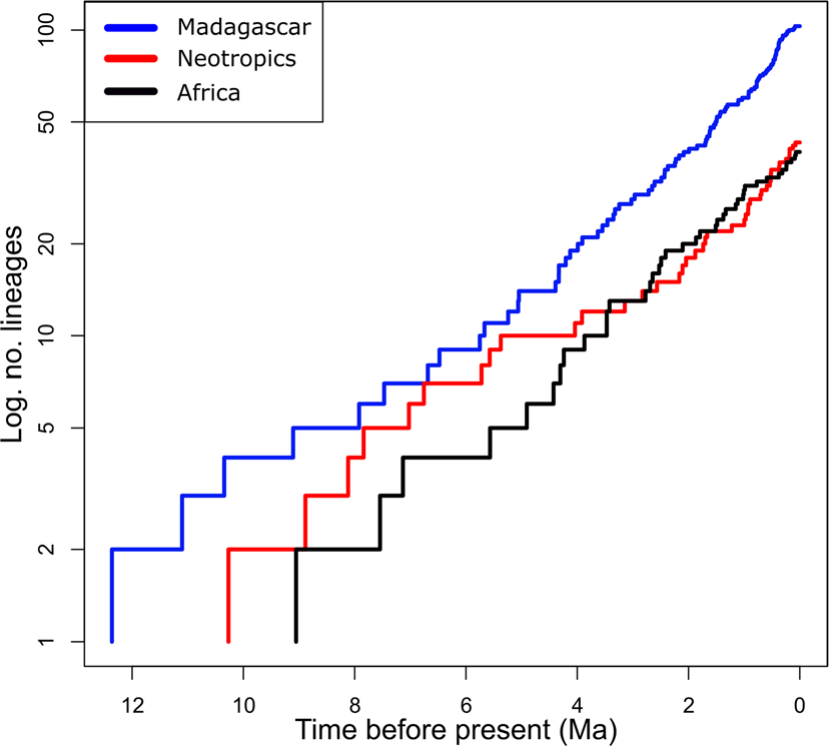
Log-lineage-through-time (LTT) plots for the Madagascan, African, and Neotropical lineages based on the chronogram depicted in Fig. 2. See Additional file 1: Figure S3 for respective LTT plots estimated from 1000 post-burn-in trees

**Fig. 4:**
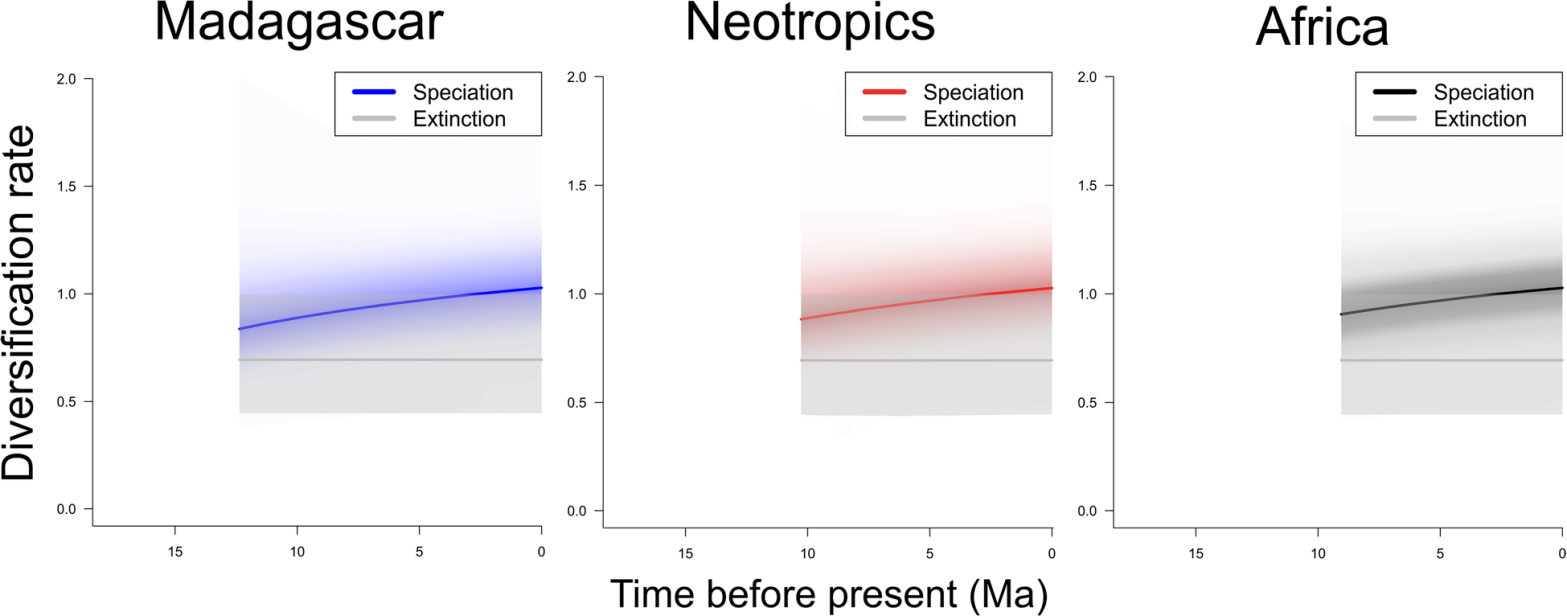
Rate-through-time plots of speciation and extinction rates of the Madagascan, African and Neotropical *Bulbophyllum* lineages as estimated by BAMM. Around each curve are the 90% credibility intervals from the posterior distribution of BAMM results

For each of the three sufficiently sampled lineages (Madagascar, Africa, Neotropics), visual inspection of their log-lineage-through-time (LTT) plots (Fig. 3), as derived from the species-level MCC chronogram (Fig. 2), suggested constant lineage accumulation through time, with no apparent slowdown towards the present (see Additional file 1: Figure S3 for respective LTT plots estimated from 1000 post-burn-in trees). Accordingly, model fitting in RPANDA (Additional file 1: Table S4) tended to favour a process of constant rates birth-death (CR-BD) diversification for both Madagascar and the Neotropics, and this model performed not significantly worse (ΔAICc = 0.79) than the best-fitting model for Africa (no extinction and constant speciation rate). Finally, for each of the three lineages, BAMM inferred no diversification-rate shift (PP values = 1.0); instead, the corresponding rate-through-time plots consistently indicated a slow but steady increase in speciation (λ) and constant extinction (*μ*) through time (see Fig. 4).

**Fig. 5:**
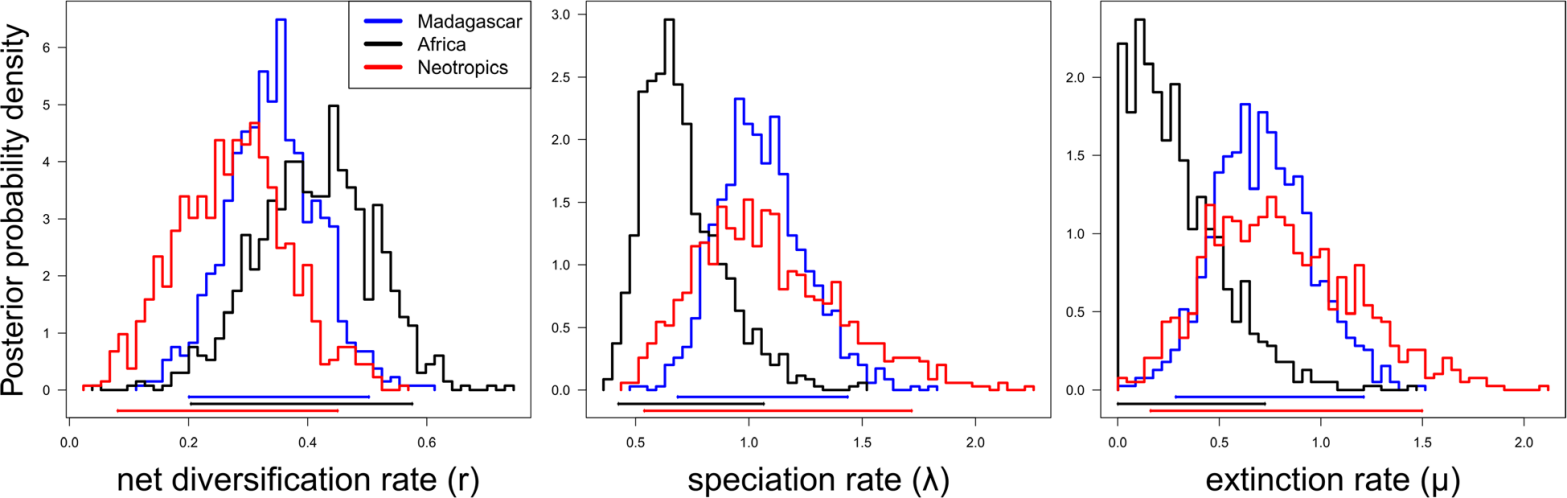
Posterior probability distribution of diversification rates of the Madagascan, African and Neotropical *Bulbophyllum* lineages as estimated by BAYESRATE. The 95% highest posterior density (HPD) intervals of parameter values are shown along the x-axes

Hence, by assuming a CR-BD model, BAYESRATE (Table 1) inferred highest posterior mean estimates of net diversification (*r*; spp./million years) for Africa (0.41), followed by Madagascar (0.34) and the Neotropics (0.27). Notably, all these estimates of *r* were consistently associated with both high speciation (*λ* = 0.72–1.08) and high extinction (*μ* = 0.29–0.80). However, because of broadly overlapping HPD intervals, none of these diversification parameters (*r*, *λ, μ*) significantly differed among the three lineages (Table 1, Fig. 5). Using Magallón and Sanderson’s [53] method, whole-clade estimates of *r* for the Asia-Pacific lineage (0.25–0.36), as variously calculated for stem/crown ages and different extinction fractions (*ε* = 0.0 or 0.9), proved to be very similar when compared to corresponding estimates for Africa (0.17–0.41), Madagascar (0.17–0.38), and the Neotropics (0.18–0.37), which in turn were also broadly consistent with the BAYESRATE results (see above; Table 1).

### Present and past (LGM) distribution of *Bulbophyllum* and suitable area estimations

**Fig. 6:**
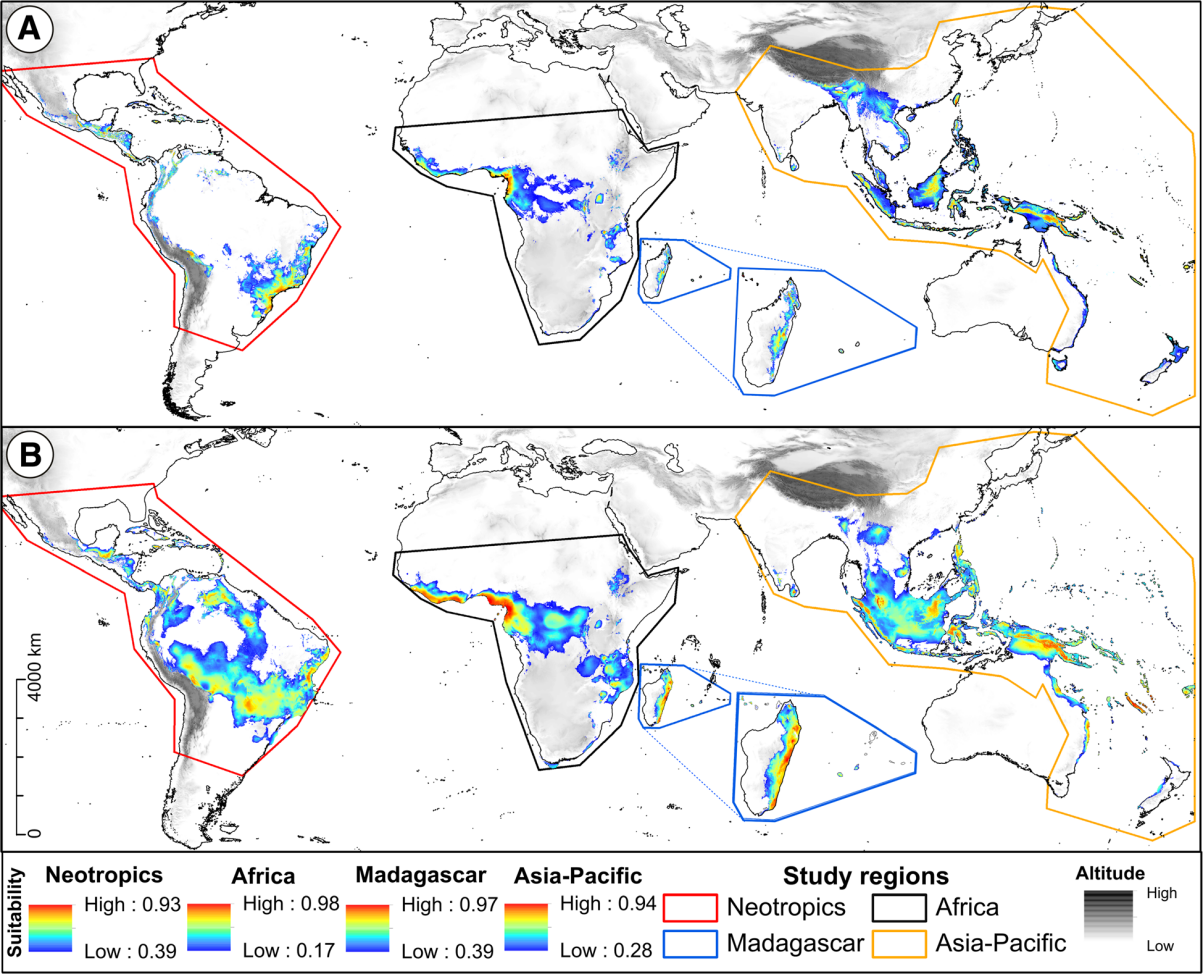
Potential distributions of the four major lineages of *Bulbophyllum* from the Neotropics, Africa, Madagascar and the Asia-Pacific region. **a** at the present (*c.* 1950–2000) and **b**) at the Last Glacial Maximum (LGM; *c.* 21,000 years ago). Ecological niche models (ENMs) were generated for each region separately using current bioclimatic variables (Additional file 1: Table S5) on the basis of extant occurrence points (Fig. 1b) of the genus using MAXENT v. 3.3.3 k. Potential distributions for the LGM are based on a consensus projection between CCSM and MIROC (see text). Predicted distribution probabilities are shown as logistic values of suitability above the region-specific maximum training sensitivity plus specificity (MTSS) thresholds. Maps were generated using ArcGIS v. 10.4.1

The regional MAXENT models for the four *Bulbophyllum* lineages had high predictive power in terms of average AUC values (± standard deviation, SD) and did not over-fit the presence data (Africa: 0.937 ± 0.018; Asia-Pacific region: 0.863 ± 0.012; Madagascar: 0.859 ± 0.025; Neotropics: 0.854 ± 0.044). Based on the stringent MTSS thresholds calculated per region (range 0.17–0.39), the current distributional predictions (Fig. 6a) covered *c.* 77.3% of all point localities used for modelling (68–88% per region), and were fairly accurate representations of the genus’ extant distribution, except for some areas modelled as (near) unsuitable while harbouring at least scattered occurrences known from current databases (e.g., northern South America; south-eastern Africa; interior of Malay Peninsula; compare Figs. 1b and 6a). By considering the number of grid cells above the mean MTSS logistic threshold (see Table 1), the Asia-Pacific region accounted for almost half of the genus’ global suitable area (*c*. 46.3%), and the Neotropics (*c*. 27.4%) and Africa (*c*. 24.9%) for about one quarter each, while Madagascar represented only a tiny fraction (*c*. 1.5%).

The Multivariate Environmental Similarity Surface (MESS) analyses (Additional file 1: Figure S5) showed a generally good agreement between the individual LGM climate models (CCSM and MIROC) in terms of similarity (viz. transferability) between the presently observed climate, used to train the MAXENT model, and the LGM projected climate (i.e. for the Neotropics, Africa, Asia-Pacific region and, to a lesser extent, Madagascar). The palaeo-distribution modelling based on the CCSM/MIROC consensus projection (Fig. 6b) suggested more extensive distribution areas of the genus in all four regions during the LGM compared with its present distribution (see Additional file 1: Figure S4 for individual climate model projections). In terms of grid cell number (Table 1), the Neotropics featured the highest increase (*c.* + 183%; due to a gain of suitable habitat especially in Coastal Brazil, Southwest Cerrado/Amazonia, as well as smaller patches in Northern Amazonia and Guiana), followed by Madagascar (+ 106%; expansion into lowlands of the north and east), Africa (+ 77%; expansion into the Congo River Basin), and the Asia-Pacific region (+ 45%; mostly due to expansion on the exposed Sundashelf). During the LGM, the largest proportion of the genus’ globally suitable area was located in the Neotropics (*c.* 41%), followed by the Asia-Pacific region (*c.* 35%), Africa (*c.* 23%) and Madagascar (*c.* 1.5%).

### Phylogenetic generalised least squares regression

The phylogenetic generalised least squares (PGLS) analyses on the *Bulbophyllum* backbone phylogeny revealed a generally strong, either marginally significant (*R*^2^ = 0.883, *P* = 0.060) or highly significant (*R*^2^ = 0.993, *P* = 0.004) relationship of, respectively, the stem and crown ages of the four lineages with their extant species numbers. There was, however, no significant effect of clade ages (stem or crown) on net diversification (*r*) rates (*R*^2^ = 0.002–0.407, *P* = 0.361–0.951), and thus regardless of which whole-clade estimator was used for the Asia-Pacific lineage (see Table 1).

Likewise, there was no evidence that the potentially suitable area of these lineages (for the present or the LGM) had any influence on their species richness (*R*^2^ = 0.030/0.004, *P* = 0.828/0.936) or on their rates of diversification (*R*^2^ *=* 0.001–0.140, *P* = 0.621–0.951).

## Discussion

Our estimated stem and crown ages of the four TRF lineages of *Bulbophyllum* (i.e. Asia-Pacific, Madagascar, Africa, Neotropics) span the early-to-late Miocene (Fig. 2, Table 1), with the genus’ crown age dated at *c.* 20.60 (16.20–25.63) Ma. Although the observed split into a Madagascan and African+Neotropical lineage is compatible with a ‘Tropical Gondwana Pattern’ (sensu Sanmartín and Ronquist [50, 55]), even our oldest estimate for the genus’ divergence time (*c*. 25.63 Ma; see above) clearly post-dates the time by which the breakup of virtually all of the individual Gondwana landmasses had begun (*c.* 80 Ma [55]; see also Givnish et al. [40]). Likewise, it is implausible to assume that *Bulbophyllum* colonized (sub) tropical regions as member of the ‘Boreotropical Flora’ around the Northern Hemisphere during the Eocene (*c.* 56–34 Ma [22]). In consequence, we discard the hypothesis of a once widespread ancestor of *Bulbophyllum* subject to successive vicariant events, as suggested by the DEC model of BIOGEOBEARS (Fig. 2). Instead, we favour the results of BAYESTRAITS (Fig. 2), which in agreement with a recent biogeographic analysis of Orchidaceae [40] reconstructed the Asia-Pacific region as the genus’ most likely ancestral area. Moreover, the node reconstructions of BAYESTRAITS are compatible with a scenario in which individual long-distance dispersal (LDD) events proceeded from the Asia-Pacific region at progressively greater distances to Madagascar, Africa and the Neotropics, respectively (as in the ‘progression rule’ of Hennig [56]), even though a far less plausible scenario (i.e. dispersal from Madagascar to Africa via the Neotropics) cannot be formally excluded.

We therefore conclude that LDD is the most likely explanation for the extant distribution of *Bulbophyllum* in virtually all TRF regions. The same conclusion has been drawn for numerous other plant groups showing similar (pan) tropical disjunctions [40, 57]. Somewhat paradoxically, however, orchids are thought to possess high dispersal capacity due to their dust-like, wind-dispersed seeds (see Gamisch et al. [49] and references therein), but direct trans-oceanic LLD events seem to be rare in this group [40, 58]. This ‘paradox of orchid dispersal’ [40], as well illustrated by the four biogeographically distinct lineages of *Bulbophyllum*, remains poorly understood but might partly reflect limits to dispersal in conjunction with missing pollinators and/or mycorrhizal fungal symbionts. On the other hand, our age estimates for these lineages (Fig. 2, Table 1) temporally match with several palaeo-events, suggesting that the progressive, dispersal-mediated diversification of *Bulbophyllum* from east to west was likely facilitated by the interplay of historical contingency and environmental change.

Accordingly, the Early Miocene radiation of the Asia-Pacific lineage [*c.* 19.12 (14.81–23.85) Ma] coincides with the rising of global temperatures after the comparatively cool Oligocene [59] and could have been further promoted by the mid-Miocene Climatic Optimum (MMCO, *c*. 17–15 Ma [59]) as well as the concomitant strengthening of the East Asian summer monsoon [60, 61]. Subsequently, now sunken islands in the western Indian Ocean, which likely existed permanently above sea level throughout the Oligocene and Miocene (Bradler et al. [62]; and references therein), could have acted as stepping-stones facilitating the genus’ dispersal from India and further Asia to the Madagascan/African region [62, 63]. In any event, the mid-to-late Miocene radiations of *Bulbophyllum* in Madagascar [*c.* 12.36 (8.23–18.84) Ma] and Africa [*c.* 9.05 (5.75–12.91) Ma] are broadly congruent with two major climatic events affecting each region separately. In Madagascar, this was the onset of heavy seasonal rainfall (*c.* 12.9–7 Ma) due to the establishment of the Indian Summer monsoon [63, 64], while at the same time (*c.* 10–8 Ma) the subsiding Congo River Basin of Central Africa experienced a general increase in humidity (‘tropicalization’) due to East African tectonic uplift [65]. Finally, the radiation of *Bulbophyllum* in South America [*c.* 10.27 (7.02–13.78) Ma] might be indirectly related to the latest stages of Andean uplift from the mid-Miocene onwards (*c.* ≤ 15–10 Ma [66]). Such geological processes not only drove climate and biotic change throughout the Amazonian Basin [67] but also affected the moisture regime and habitat diversity of the coastal Atlantic Forest, especially through the (close to present) re-organization of the Amazon River in the Late Miocene/Early Pliocene [68, 69]. Hence, as recently postulated for Amazonian TRF tree communities [70], such environmental instability might have also created opportunities for both the successful immigration and radiation of Neotropical *Bulbophyllum*. Overall, these results suggest a major role for early-to-late Miocene changes in climate and/or geology in shaping the genus’ pantropical distribution and initial diversification.

There are three major lines of argument to suggest that disparities in species richness amongst the four TRF lineages (Madagascar: 210 spp.; Africa: 80; Neotropics: 94; Asia-Pacific region: 1564) are neither explicable by differential levels of ‘carrying capacity’ (i.e. constraints on diversification due to spatial-ecological limits) nor differences in diversification rate, but most likely result from a time-for-speciation (i.e. clade age) effect (see Introduction). First, each of the sufficiently sampled lineages (Madagascar, Africa, Neotropics) evolved under a CR-BD process since their onset of diversification (mid-to-late Miocene; Fig. 2), as variously inferred from (1) the shapes of their LTT plots (Fig. 3, Additional file 1: Figure S3); (2) model fitting in RPANDA (Additional file 1: Table S4); and (3) rate-through-time plots (BAMM Fig. 4). Hence, none of these lineages showed an apparent slowdown of diversification over time, as would be expected under the ‘carrying capacity’ hypothesis [36]. Secondly, using BAYESRATE, we found no significant differences among the net diversification rates of these three lineages (*r* = 0.27–0.41; Table 1, Fig. 5). In addition, for the Asia-Pacific lineage, all method-of-moments estimators [53] indicated similar net diversification rates (*r* = 0.25–0.36), again falling within the 95% HPD intervals of the corresponding BAYESRATE analysis (Table 1). Hence, even though this latter lineage warrants further estimates of net diversification based on branching times, the present results provide no evidence that species-rich TRF lineages of *Bulbophyllum* diversify more rapidly. Finally, given this latter outcome, it is not unexpected that our PGLS analyses of the combined dataset (including all four lineages) failed to detect any significant influence of clade ages (stem or crown) or potentially suitable areas (for the present or the LGM) on these net diversification rates (*R*^2^ = 0.002–0.407, all *P* ≥ 0.361). In addition, we found no significant relationship between our regional estimates of present and past climatic niche space and the number of extant species in each of the four lineages (both *R*^2^ ≤ 0.030, *P* ≥ 0.828), and thus no spatial-bioclimatic constraints on species richness. Instead, disparities in species richness amongst the four lineages appeared to be primarily influenced by stem/crown group ages (*R*^2^ = 0.883/0.993, *P* = 0.060/0.004). Overall, these findings suggest a pivotal role for a clade age effect in generating species richness disparities among the four TRF lineages of *Bulbophyllum* [27, 29, 71]. Moreover, with regard to those sufficiently sampled (Madagascar, Africa, Neotropics), our data appear sufficiently robust to exclude major constraints of present or past (LGM) climatic niche space on diversification; rather, all three lineages are apparently still in their ‘growth phase’ (e.g. Fig. 3 and 4; [26, 72]). This is perhaps most remarkable in the case of Madagascar, which has probably never provided more than only a tiny fraction of the genus’ total suitable area (*c.* 1.4 and 1.3% at present and the LGM, respectively; Table 1).

For *Bulbophyllum* it would appear then that present or past spatial-bioclimatic limits on diversity are either absent or very high in each of the three TRF regions [2, 25] and/or that the time period available to accumulate species has been insufficient to reach those limits [73]. However, a third, mutually non-exclusive explanation is that such limits have not been reached yet because the diversification of these three lineages is regulated by high species turnover, meaning that species are formed and replacing each other at a high pace [19]. This hypothesis gains support from the fact that the Madagascan, African and Neotropical lineages feature high rates of both speciation (*λ* = 0.70–1.08) and extinction (*μ* = 0.29–0.80; Table 1, Fig. 5), resulting in net diversification rates (*r* = 0.27–0.41; see above) that are only low-to-moderate when compared to other, often much faster plant radiations in the tropics (*r* > 1.0 [74, 75]) or elsewhere [30–32]. Why lineages diversify under such high speciation and extinction rates has generally received little theoretical/empirical attention but is commonly thought to reflect severe environmental change in climate and/or habitat conditions [19, 23, 76, 77].

As none of these three lineages (Madagascar, Africa, Neotropics) provides evidence of significant diversification-rate shifts (see above), one might conclude that environmental change during the Quaternary (e.g., [69, 78]) had no important role in their *temporal* course of diversification (e.g., [54]). Hence, a possible explanation for much of their high species turnover could be sought in various intrinsic features commonly invoked to foster rapid population and species turnover in tropical orchids by conferring the potential to seize ecological opportunity while increasing the risk of extinction (e.g., epiphytism, specialization on pollinators and mycorrhizal fungi, resource-limited reproduction, dispersal by wind [51, 58, 79]). However, there is presently little evidence to suggest that high species turnover is a common feature of tropical orchids (e.g., Givnish et al. [51]; *Dendrobium* [80]; but see Neotropical Pleurothallidinae [75]). Also, we caution that failure of our modelling methods (RPANDA, BAMM) to detect diversification-rate shifts does not imply that the null hypothesis of constant-rate evolution is ‘true’, but only that there is insufficient evidence to reject this hypothesis, as probably best achieved with larger-sized lineages (≥ 300 taxa; cf. [81]).

In any event, constant-rate evolution in the above *Bulbophyllum* lineages does not necessarily rule out a potential influence of Quaternary environmental instability on their *mode* of diversification viz. the origination and extinction of species (sensu Matos-Moraví [54]). This hypothesis gains support from two lines of argument. First, our molecular dating provides sufficiently robust evidence that most extant species, at least in Madagascar, Africa and the Neotropics, are of Quaternary age (Fig. 2). And second, our ENM projections for the present and the LGM indicate that all four lineages (including Asia-Pacific) experienced dramatic changes in range size in the form of glacial expansions and inter-/postglacial contractions (compare Figs. 6a vs. b, Table 1). Both events could have facilitated the emergence of new species through, respectively, divergent ecological adaptation and vicariance, while range contractions likely caused species extinction through habitat loss (e.g., [65, 82]. Nevertheless, when taken on balance, the present data seem to suggest that high species turnover in *Bulbophyllum* is more likely a consequence of intrinsic features rather than repeated range shifts during the Quaternary, their potential role in speciation/extinction mechanisms notwithstanding.

Finally, and independent of any diversification scenario, the global signature of glacial range expansion in *Bulbophyllum* (Fig. 6b) deserves brief comment as it stands in direct contrast to classical theories of Quaternary diversification in the tropics (e.g., [11, 54, 83]). According to those ‘glacial refuge’ models, the range sizes of TRF-dwelling taxa contracted during glacial periods of aridity, whether proposed for the Neotropics [83], tropical Africa [10, 12, 13], Madagascar [84], or the Asia-Pacific/Sundaland region [1]. Why *Bulbophyllum* expanded during glacials could relate to several taxon-specific traits conferring high tolerance to drought and water stress (e.g., water-storing pseudobulbs; thick, evergreen leaves minimizing transpiration; water-saving crassulacean acid metabolism/CAM [43, 49]). In addition, there is increasing evidence from palaeo-data and/or phylogeographic studies that the climate of several areas where *Bulbophyllum* expanded at the LGM was still suitable (e.g., relatively humid) to sustain TRF communities over the last glacial cycles, whether in Africa (e.g., Congo River Basin [65]; Niger River Catchment, Eastern Arc Mountains of Tanzania [61]; coastal West Africa [14]), Madagascar (northern and eastern parts [18]), the Neotropics (Brazilian Atlantic Forest [85, 86]) or the Asia-Pacific region (Sundaland [17, 87]). Hence, together with these recent studies, the present ENM data challenge the long-held notion that TRFs mostly fragmented during glacial periods. In turn, this would suggest that the currently contracted TRF ranges of *Bulbophyllum* are in a ‘refugial stage’ of likely high vulnerability (see also [17]).

## Conclusions

The present study identifies *Bulbophyllum* as an ideal model system of testing fundamental hypotheses about evolutionary, biogeographic and diversification processes shaping species richness disparities amongst the Earth’s four major TRF regions as well as the range dynamics of these forest biomes in response to past (e.g., Quaternary) climate change. Our molecular dating and biogeographic analyses of this orchid mega-genus suggest an early-to-late Miocene scenario of ‘out-of-Asia-Pacific’ origin and progressive (east-to-west) dispersal-mediated diversification, resulting in three additional radiations in Madagascar, Africa and the Neotropics, respectively. Moreover, our results indicate that current species richness disparities amongst these four TRF lineages is largely a function of clade age rather than a result of among-lineage variation in net diversification rate or carrying capacity (viz. spatio-bioclimatic limits). The constantly high species turnover of the Madagascan, African and Neotropical lineages is likely more generally influenced by various intrinsic features conferring high population/species turnover in tropical orchids than by extrinsic factors, such as Quaternary environmental change; nonetheless, repeated range shifts during this latter period could still have played an influential role in the origination and extinction of *Bulbophyllum* species in all four TRF regions. Clearly, the validity of the above inferences requires further testing as they largely rest on a single-marker (ITS) phylogeny with insufficient sampling of the Asia-Pacific region. Nonetheless, despite these limitations, our study is the first to examine the range-wide diversification dynamics of *Bulbophyllum*. As such, it should motivate further (e.g., phylogenomic and ecological) research not only in this but also in other pantropical TRF taxa. This should yield a better understanding of how evolutionary processes as well as past and current environmental conditions drive tropical biodiversity and account for regional differences in species richness patterns on a global scale.

## Methods

### Phylogenetic taxon sampling and molecular dating

This study builds upon a time-calibrated ITS (ITS1 + 5.8S + ITS2) phylogeny of *Bulbophyllum* (262 spp./266 accessions) previously employed for dating Madagascan ‘clade C’ [48] (see Additional file 8 in Gamisch et al. [48]). After removing still unidentified accessions (44 in total), we supplemented this previous dataset with altogether 110 GenBank-derived ITS sequences, representing 98 species of *Bulbophyllum* (Madagascar: 94; Neotropics: 4) and six species each of its successive sister genera from the Asia-Pacific region, i.e. *Dendrobium* Sw. and *Epigeneium* Gagnep. [51]. In sum, this overall enlarged ITS dataset represents *c.* 16.4% of the total diversity of *Bulbophyllum* (320/1948 spp. [41]), including sufficient samples from Madagascar (103 out of 210 spp.; 49.04%), Africa (40/80; 50%) and the Neotropics (43/94; 45.74%), but less so from the Asia-Pacific region (132/1564; 8.4%), plus the 12 outgroup species. All 332 ITS sequences, including 79 previously unpublished ones of Gamisch et al. [48], are available from GenBank (see Additional file 1: Table S1 for accession numbers and vouchers/references).

Sequences were edited manually and aligned using the ClustalW algorithm with default settings in GENEIOUS v. 10.2.3 [88]. The final alignment consisted of 765 nucleotide sites, 466 of which were parsimony informative. The best fitting model of nucleotide substitution (GTR + G + I) was identified using the Bayesian information criterion (BIC) as implemented in IQ-TREE v. 1.6.2 (http://iqtree.cibiv.univie.ac.at; [89]). Absolute divergence times and phylogenetic relationships within *Bulbophyllum* were estimated in BEAST v. 1.8.4 [90] and modelled with a normal prior distribution [91] (see also Additional file 1: Figure S1): (1) the crown age of Dendrobieae (mean ± SD: 30.17 ± 3.480 Ma), following Gamisch et al. [48]; (2) the crown age of *Dendrobium* (28.35 ± 1.649 Ma), following Xiang et al. [80]; and (3) the divergence time between *Bulbophyllum* and *Epigeneium* (20.55 ± 3.998 Ma), following Givnish et al. [51]. Constraints in topology were applied to match the previously inferred topology of Dendrobieae [51], i.e. (*Dendrobium* (*Bulbophyllum, Epigeneium*)). A relaxed molecular clock analysis with uncorrelated log-normal model was used [92], as also validated by a coefficient of variation of 0.67 (i.e. > 0.10; [93]). The tree speciation prior followed a CR-BD process [94], and one Markov chain Monte Carlo (MCMC) run was performed on the CIPRES Science Gateway portal [95] for 10^8^ generations, sampling every 10,000th step. TRACER v. 1.5 (http://tree.bio.ed.ac.uk/software/tracer/) was used to confirm that all parameters had large enough effective sample sizes (ESS > 210) after the removal of 10% as burn-in.

### Ancestral area reconstructions

The large-scale biogeographic history of *Bulbophyllum* was reconstructed using the BEAST-derived species level maximum clade credibility (MCC) chronogram with each species coded according to its presence in one of the four TRF regions, following Sieder et al. [41] (i.e. A: Asia-Pacific; B: Madagascar; C: Neotropics; D: Africa; see also insert map of Fig. 2). Ancestral area states were reconstructed for eight nodes of interest (root, stem and crown nodes of the four lineages) using the Bayesian Binary MCMC framework of the submodule MULTISTATE of BAYESTRAITS v. 1.0 [96]. For three key nodes (labelled I–III in Fig. 2) statistical support for estimated ancestral areas was evaluated against alternative states using 2 logarithmic Bayes Factor (BF) values based on marginal likelihoods as calculated in TRACER (see Additional file 1: Table S2). Following Kass and Raftery [97], evidence for a constrained state (compared to a constrained alternative state A, B, C or D) was considered to be ‘weak’ (BF value = 0–2), ‘positive’ (2–5), ‘strong’ (5–10), or ‘decisive’ (> 10). Each MCMC analysis was run for 5.05 × 10^6^ generations, using a reversible-jump hyperprior with an exponential prior (uniform distribution on the interval 0 to 30), and a burn-in of 5 × 10^4^ generations. As an alternative approach, we also used a likelihood-based framework in BIOGEOBEARS v. 0.2.1 [98], assuming the dispersal-extinction-cladogenesis (DEC) model of range evolution [99], as implemented in RASP v. 4.0 [100]. This model was selected when tested against two alternative models (DIVALIKE, BAYAREALIKE) based on size-corrected AIC (AICc) values (see Additional file 1: Table S3). Models were considered comparable if ΔAICc was < 2.0 [101].

### Diversification analyses

For each sufficiently sampled lineage (Madagascar, Africa, Neotropics), we used multiple approaches to explore their diversification dynamics. First, we graphically assessed their rates of net diversification, *r* [i.e. speciation (*λ*) – extinction (*μ*)], through LTT plots derived from, respectively, the species-level MCC chronogram (Fig. 2) and 1000 post-burn-in trees, using GEIGER v. 2.0.6 [102]. Second, we fitted a complex set of nine diversification models to each lineage, using the maximum likelihood fit_bd function of RPANDA v. 1.3 [103] (see Results and Additional file 1: Table S4) and ΔAICc (< 2.0) for model selection (see above). After a burn-in of 10% of iterations we used TRACER to verify convergence of each run and each parameter (ESS > 630). Third, to further test for shifts in diversification rate (*λ*, *μ*) within each lineage, we used BAMM v. 2.5.0 (http://bamm-project.org), which explores multiple models (‘configurations’) of diversification-rate heterogeneity using reversible-jump MCMC simulations [104]. For each dataset, we performed one MCMC run with 10^6^ iterations and a sampling frequency of 1000 under the default prior assumption of a single expected rate shift. Starting priors for *λ* and *μ* etc. were obtained using the setBAMMpriors function in BAMMTOOLS v. 2.1.6 [105]. Post-analysis and visualization of rate-through-time plots (*λ*, *μ*) were carried out using BAMMTOOLS. Finally, for each of the three lineages, we used BAYESRATE v. 1.6.3 beta [52] to estimate posterior mean values and 95% HPD intervals of all diversification parameters (*r*, *λ*, *μ*) under a CR-BD model (*μ* > 0), as selected by RPANDA (see Results). Program settings were similar to above (MCMC run per dataset, 10^7^ iterations, sampling frequency 1000). As incomplete taxon sampling can lead to biased estimates of diversification rates on molecular phylogenies (e.g. Rabosky et al. [104]), we analytically accounted for missing species in our likelihood (RPANDA) and Bayesian (BAMM, BAYESRATE) analyses using lineage-specific sampling fractions (i.e. Madagascar: 103 out of 210 spp.; 49.04%; Africa: 40/80, 50%; Neotropics: 43/94; 45.74%).

For the less well-sampled Asia-Pacific lineage (132/1564 spp.), diversification parameters could not be calculated in BAYESRATE due to insufficient information on branching times. Instead, we used the ‘methods-of-moment’ estimator of Magallón and Sanderson [53], as implemented in LASER v. 2.4.1 [106], which requires only clade age (either stem or crown) and clade size (extant species number) to estimate *r* under different values of relative extinction (*ε* = *μ*/*λ*). Following standard practice, we assumed two values of *ε* (zero and high relative extinction: *ɛ* = 0.0 and 0.9), even though different values usually have relatively little impact on the results [107]. For comparison, we also applied this ‘whole-clade’ method to the stem and crown ages of the three other lineages.

### Present and past (LGM) ecological niche modelling and suitable area estimations

The global distribution of *Bulbophyllum* has never been assessed using occurrence data points (but see the outline map of Pridgeon et al. [42]). We therefore generated a point locality map for the entire genus (Fig. 1b) based on data from the Global Biodiversity Information Facility (GBIF), herbarium collections and literature (see Supplementary Methods for details). For the ENM analyses, each of the four regional datasets of occurrence data (Asia-Pacific, Madagascar, Africa, Neotropics) was subsequently pruned to one random accession per species to account for both imbalanced numbers of point localities per species (range: 1–458; mean ± SD: *c.* 8 ± 22.38) and environmentally biased sampling (near roads, towns, etc. [108]). This pruning was then repeated 10 times, resulting in a maximum of 10 random localities per species. All unidentified accessions of a given region were considered as a single unit and treated in the same way.

Based on these 40 locality datasets, current distribution models were developed separately for each region using 19 bioclimatic data layers for the present (*c*. 1950–2000) as available from the WorldClim database v. 1.4 [109] at 2.5 arc-min resolution (4.65 × 4.65 = 21.623 km^2^ at the equator). Highly redundant climatic variables (Pearson’s correlation > 0.9), as identified by ENMTOOLS v. 1.4.4 [110], were removed to avoid potential over-fitting (see [111] and references therein). Based on the remaining variables (nine to 11 per region; see Additional file 1: Table S5), the genus’ current potential distribution was modelled in MAXENT v. 3.3.3 k [112] for each region and locality dataset separately, using 10 bootstrap replicates and 75% of the localities to train the model, while allowing for multiple presence records of different species within the same grid cell. Model performance was evaluated using receiver operating characteristic (ROC) analyses in MAXENT. Values under the area of the ROC curve (AUC) between 0.7 and 0.9 indicate good fit (see [111] and references therein).

The established models were then projected onto conditions of the LGM (2.5 arc-min resolution) based on either the Community Climate System Model (CCSM4) or the Model for Interdisciplinary Research on Climate (MIROC-ESM; both available from WorldClim v. 1.4). This was done by restricting (‘clamping’) the projected variables within the range of values encountered during model training under current conditions [112]. We also applied the MESS method of Elith et al. [113], implemented in MAXENT, to measure, for any grid cell, the similarity between the LGM projected climate and the current observed climate used to train the model, whereby positive and negative MESS scores indicate analogue and no-analogue climates, respectively.

Based on the 10 locality datasets per region (see above), and their respective CCSM and MIROC projections for the LGM, consensus predictions were calculated in ArcGIS v. 10.4.1 (ESRI, Redland, CA) for each region (Fig. 6a, Additional file 1: Figure S4). In addition, a consensus LGM prediction map (Fig. 6b) was generated by averaging over both palaeo-projections (CCSM and MIROC). Finally, for each region and time period (present, LGM), we quantified the extent of potentially suitable area viz. climatic niche as the number of grid cells above the mean ‘maximum training sensitivity plus specificity’ (MTSS) logistic threshold, which has been shown to outperform other threshold options available [114]. We caution that our ENM projections only consider climatic (rather than additional abiotic or even biotic) variables, assuming they sufficiently represent conserved niche requirements of the four *Bulbophyllum* lineages [49, 115]. However, despite these limitations, similar approaches have provided reasonably accurate inferences about the range dynamics of (sub) tropical and temperate biota at various spatial and taxonomic scales (clades, species, populations) over the last glacial cycle(s) (Araújo et al. [116] and references therein).

### Phylogenetic generalised least squares

Based on the *Bulbophyllum* backbone phylogeny (with each major lineage collapsed into a single terminal unit), we used PGLS [117], as implemented in CAPER v. 0.5.2 [118], to test for the influence of clade age or potentially suitable area (for the present and the LGM) on net diversification (*r*) as well as patterns of regional species richness. In contrast to standard regressions, PGLS takes into account phylogenetic autocorrelation, i.e. the degree to which species are related [118]. Values of *r* for the Madagascan, African and Neotropical lineages were derived as mean posterior estimates from BAYESRATE. For the Asia-Pacific lineage we used all four whole-clade estimates [53], as calculated for stem or crown ages under different extinction scenarios (*ɛ* = 0.0 and 0.9; see above). Estimates of area and species richness were log-transformed prior to analysis [29].

## Additional file

Additional file 1: **Supplementary Methods**. **Figure S1.** Ages of the BEAST-derived species-level maximum clade credibility (MCC) chronogram. **Figure S2.** BEAST-derived MCC chronogram with all numerical posterior probability (PP) values. **Figure S3.** LTT plots of 1000 trees sampled from the posterior distribution of the BEAST analyses. **Figure S4.** Current distribution models of the four *Bulbophyllum* lineages projected onto climatic conditions of the LGM derived from CCSM and MIROC, respectively. **Figure S5.** Representative Multivariate Environmental Similarity Surface (MESS) analyses of individual CCSM and MIROC models for LGM climatic conditions. **Table S1.** GenBank accession numbers and vouchers/references for 332 nrDNA (ITS) sequences of *Bulbophyllum* (320), *Dendrobium* (6) and *Epigeneium* (6), subdivided by geographic region. Note, this list includes 253 accessions obtained from GenBank (NCBI) plus 79 previously unpublished sequences of Gamisch et al. [48] (the latter marked in bold). **Table S2.** Statistical support for estimated ancestral areas, using BAYESTRAITS. **Table S3.** Biogeographical model fitting, using BIOGEOBEARS. **Table S4.** Diversification models fitted to the crown groups of Madagascan, African, and Neotropical *Bulbophyllum*, using RPANDA. **Table S5.** Bioclimatic variables used for the ecological niche modelling. (PDF 7437 kb)

## Abbreviations

AIC: Akaike information criterion
AUC: Area under the curve
BF: Bayes factor
BIC: Bayesian information criterion
CAM: Crassulacean acid metabolism
CCSM: Community Climate System Model
CR-BD: Constant rates birth-death
DEC: Dispersal-extinction-cladogenesis
ENM: Ecological niche modelling
ESS: Effective sample sizes
GBIF: Global Biodiversity Information Facility
HPD: Highest posterior density
ITS: internal transcribed spacer
LDD: Long-distance dispersal
LGM: Last Glacial Maximum
LTT: Log-lineage-through-time
MCC: Maximum clade credibility
MCMC: Markov chain Monte Carlo
MESS: Multivariate Environmental Similarity Surface
MIROC: Model for Interdisciplinary Research on Climate
MMCO: Mid-Miocene Climatic Optimum
MTSS: Maximum training sensitivity plus specificity
PGLS: Phylogenetic generalised least squares
ROC: Receiver operating characteristic
SD: Standard deviation
TRF: Tropical rainforest
