# Supplementary material for: Clade-age-dependent diversification under high species turnover shapes species richness disparities among tropical rainforest lineages of Bulbophyllum (Orchidaceae)
Source: BMC Evol Biol. 2019 Apr 24;19:93. doi: 10.1186/s12862-019-1416-1 (PMC6480529; doi:10.1186/s12862-019-1416-1)
Supplement: Supplementary file 1 — Supplementary Methods. Figure S1. Ages of the BEAST-derived species-level maximum clade credibility (MCC) chronogram. Figure S2. BEAST-derived MCC chronogram with all numerical posterior probability (PP) values. Figure S3. LTT plots of 1000 trees sampled from the posterior distribution of the BEAST analyses. Figure S4. Current distribution models of the four Bulbophyllum lineages projected onto climatic conditions of the LGM derived from CCSM and MIROC, respectively. Figure S5. Representative Multivariate Environmental Similarity Surface (MESS) analyses of individual CCSM and MIROC models for LGM climatic conditions. Table S1. GenBank accession numbers and vouchers/references for 332 nrDNA (ITS) sequences of Bulbophyllum (320), Dendrobium (6) and Epigeneium (6), subdivided by geographic region. Note, this list includes 253 accessions obtained from GenBank (NCBI) plus 79 previously unpublished sequences of Gamisch et al. [48] (the latter marked in bold). Table S2. Statistical support for estimated ancestral areas, using BAYESTRAITS. Table S3. Biogeographical model fitting, using BIOGEOBEARS. Table S4. Diversification models fitted to the crown groups of Madagascan, African, and Neotropical Bulbophyllum, using RPANDA. Table S5. Bioclimatic variables used for the ecological niche modelling. (PDF 7437 kb) [file 12862_2019_1416_MOESM1_ESM.pdf]

Supplementary Information

Clade-age-dependent diversification under high species turnover shapes species richness disparities among tropical rainforest lineages of *Bulbophyllum* (Orchidaceae)

Alexander Gamisch and Hans Peter Comes

Department of Biosciences, University of Salzburg, A-5020, Austria

INCLUDING: Supplementary Methods, Figure S1-S5, Tables S1–S5

Supplementary Methods

Occurrence data and Map generation Occurrence data were collected from the Global Biodiversity Information Facility (GBIF) [(18th April 2016) GBIF Occurrence Download http://doi.org/10.15468/dl.fcg7zi] and supplemented by additional geo-referenced records taken from both the literature (Averyanov 2010, 2012; Vermeulen and Tsukaya 2011; Karuppusamy and Ravichandran 2013; Chowlu *et al.* 2014; Roy *et al.* 2014; Verma *et al.* 2014; Van Duy and Averyanov 2015; Averyanov *et al.* 2016, 2017; Juswara *et al.* 2016) and herbarium collections (see Gamisch 2014 for full details). Intra-specific locality duplicates were excluded. The resulting map generated in ArcGIS v. 10.4.1 (Fig. 1B) comprised 7,528 presence records (6,944 localities), representing 934 species [*c.* 47.9% of the total; Madagascar: 73% (154/210); Africa: >100% (106/80); Neotropics: 68% (64/94); Asia-Pacific region: 39% (610/1,564); Sieder *et al.* 2009] plus 388 accessions of unknown species identity (Madagascar: 144; Africa: 91; Neotropics: 16; Asia-Pacific: 137).

Supplementary References

Averyanov, L. V. Species of orchids (Orchidaceae) newly recorded to the flora of Vietnam. *Taiwania* **55**, 1–7 (2010).

Averyanov, L. V. New orchid taxa and records in the flora of Vietnam. *Taiwania* **57**, 127–152 (2012).

Averyanov, L. V. *et al.* New species of orchids (Orchidaceae) in the flora of Vietnam. *Taiwania* **61**, 319–354 (2016).

Averyanov, L. V., Nguyen, K. S., Nong, V. D., Nguyen, V. C., Truong, B. V. & Maisak, T. V. *Bulbophyllum* sect. *Hirtula* in eastern Indochina. *Taiwania* **62**, 1–23 (2017).

Chowlu, K., Mahar, K.S., Sharma, H.B., Ngapui, A., Rao, N. & Vij, S. P. *Bulbophyllum dickasonii* Seidenf. (Orchidaceae): a new record for India with a complete morphological description. *Kew Bull.* **69**, 9495 (2014).

Duy, N. V. & Averyanov, L. V. *Bulbophyllum bidoupense* and *Schoenorchis hangianae* – new species of orchids (Orchidaceae) from southern Vietnam. *Phytotaxa* **213**, 113–121 (2015).

Gamisch, A. *The evolution of auto-pollination in tropical orchids: Morphological experimental and phylogenetic studies in Bulbophyllum THOUARS from Madagascar. PhD thesis.* (University of Salzburg, Salzburg, 2014).

Juswara, L., Schuiteman, A. & Droissart, V. Four new orchid species from the Lengguru fold belt, West Papua, Indonesia. *PhytoKeys* **61**, 47–59 (2016).

Karuppusamy, S. & Ravichandran, V. A New Orchid of the Genus *Bulbophyllum* (Orchidaceae) from Western Ghats of Southern India. *TAPROBANICA* **5**, 120–123 (2013).

Roy, D., Barbhuiya, H. A., Talukdar, A. D. & Sinha, B. K. *Bulbophyllum mahabendrae* (Orchidaceae: Epidendroideae), a new species from Meghalaya, India. *Phytotaxa* **164**, 291–295 (2014).

Sieder, A., Rainer, H. & Kiehn, M. *CITES Orchid Checklist. Volume 5. CITES checklist for Bulbophyllum and allied taxa (Orchidaceae).* (Kew Publishing, Kew, UK, 2009).

Verma, D., Barbhuiya, H. A. & Lavania, S. *Bulbophyllum cherrapunjeensis* (Orchidaceae), a new species from Meghalaya, India. *Phytotaxa* **156**, 298–300 (2014).

Vermeulen, J. J. & Tsukaya, H. An assumed rheophytic orchid: *Bulbophyllum rheophyton* n. sp., from Borneo. *Plant Syst. Evol*. **293**, 71–73 (2011).

Supplementary Figures

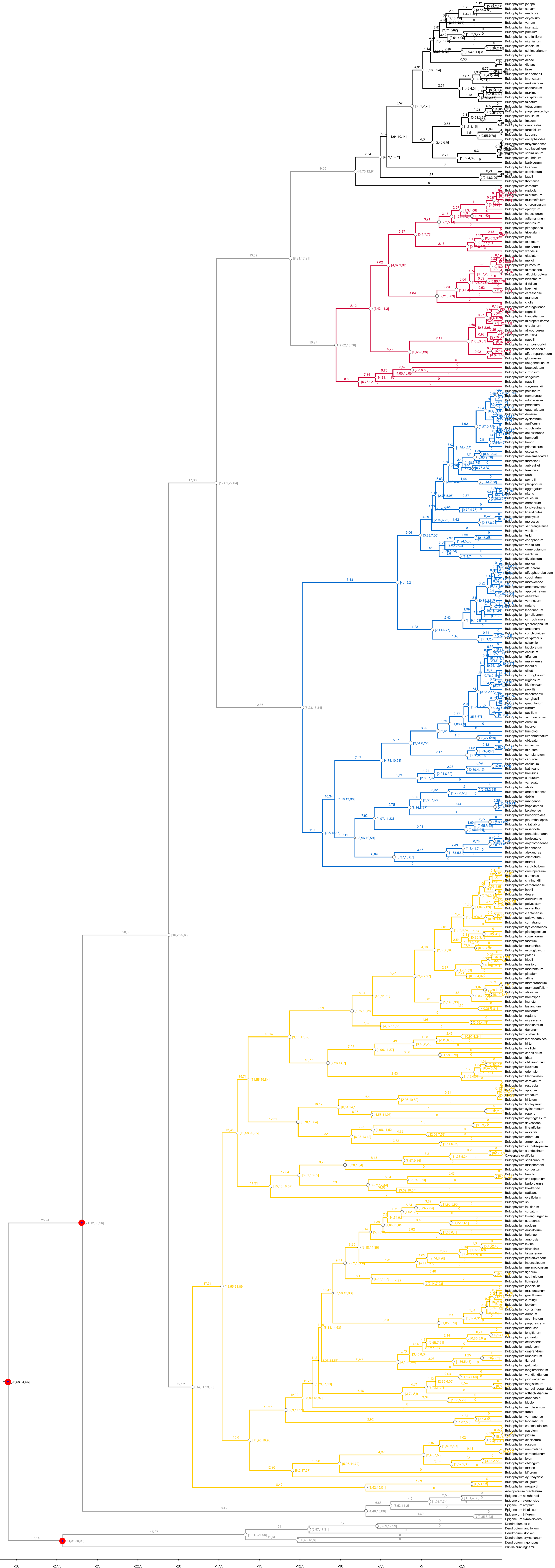

-30
-27,5
-25
-22,5
-20
-17,5
-15
-12,5
-10
-7,5
-5
-2,5

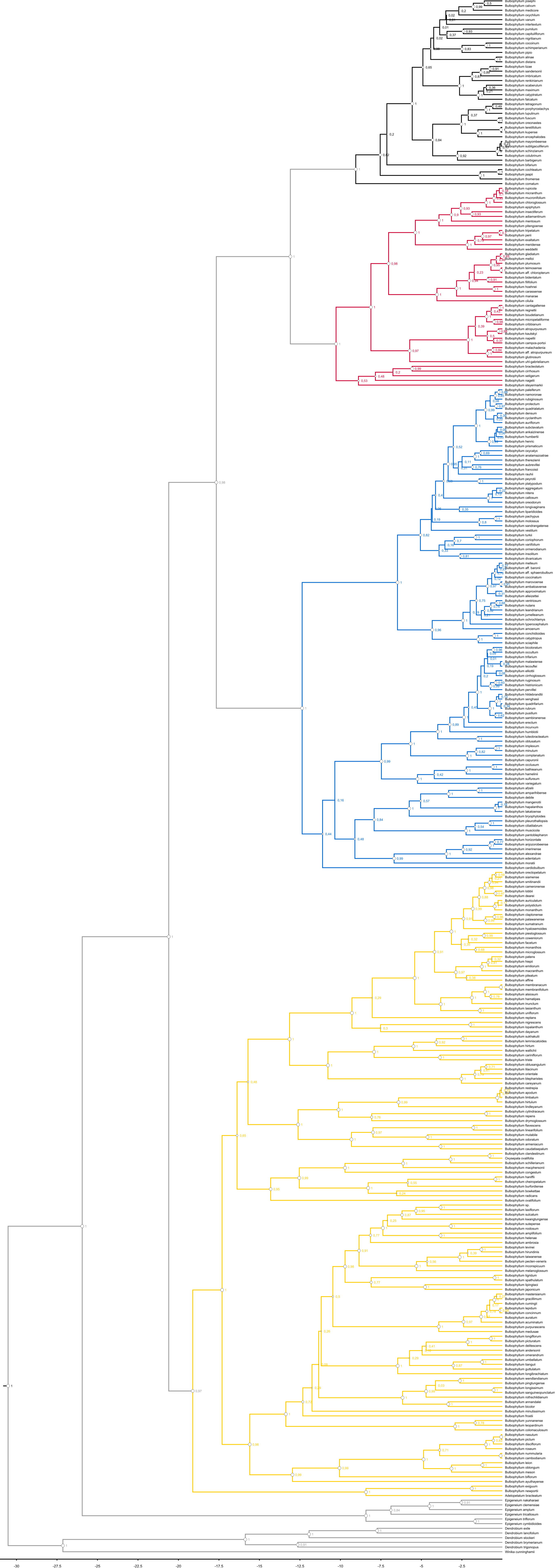
-30
-27.5
-25
-22.5
-20
-17.5
-15
-12.5
-10
-7.5
-5
-2.5

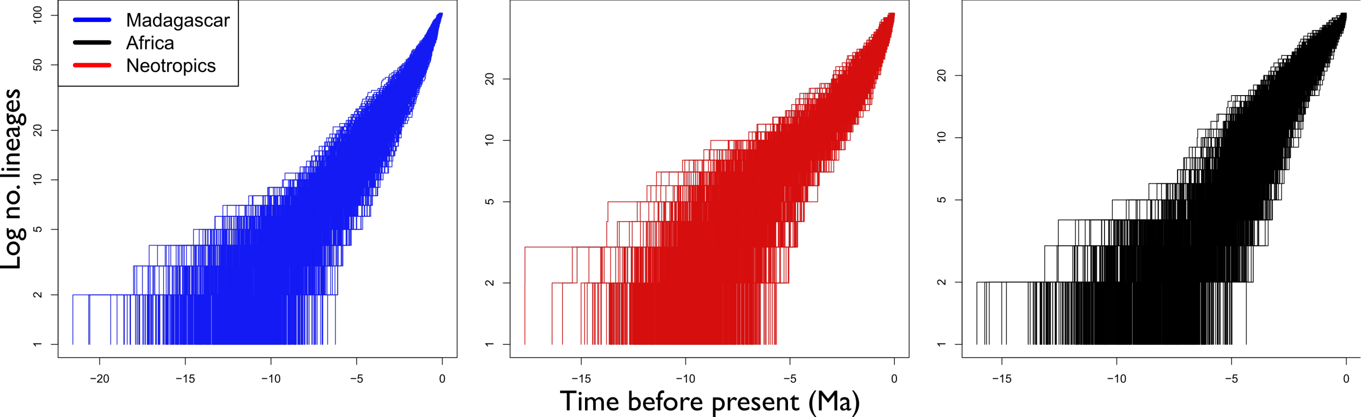
Madagascar
Africa
Neotropics
Log no. lineages
100
50
20
10
5
2
1
−20
−15
−10
−5
0
Time before present (Ma)

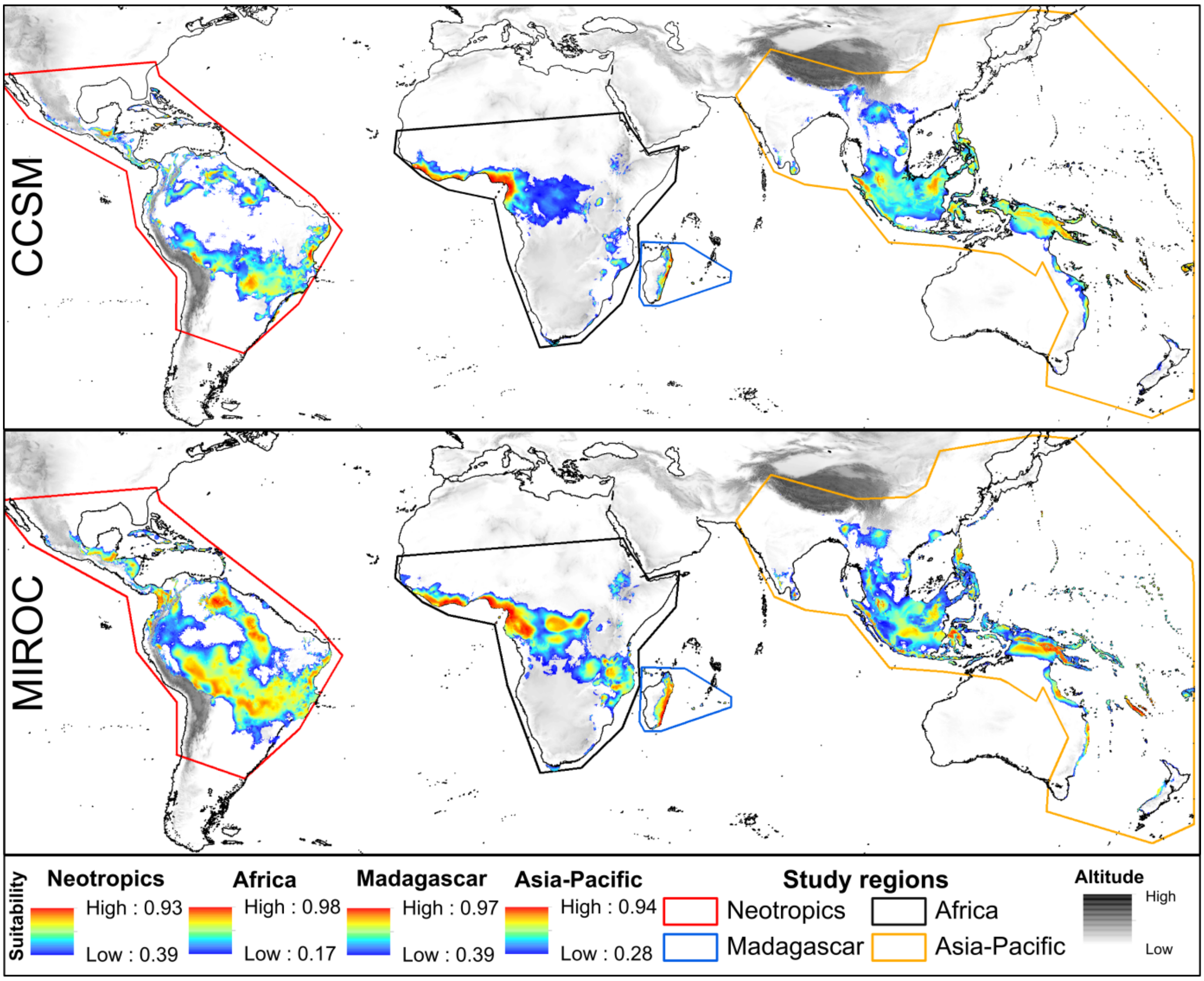
CCSM
MIROC
Suitability
Neotropics
High : 0.93
Low : 0.39
Africa
High : 0.98
Low : 0.17
Madagascar
High : 0.97
Low : 0.39
Asia-Pacific
High : 0.94
Low : 0.28
Study regions
Neotropics
Africa
Madagascar
Asia-Pacific
Altitude
High
Low

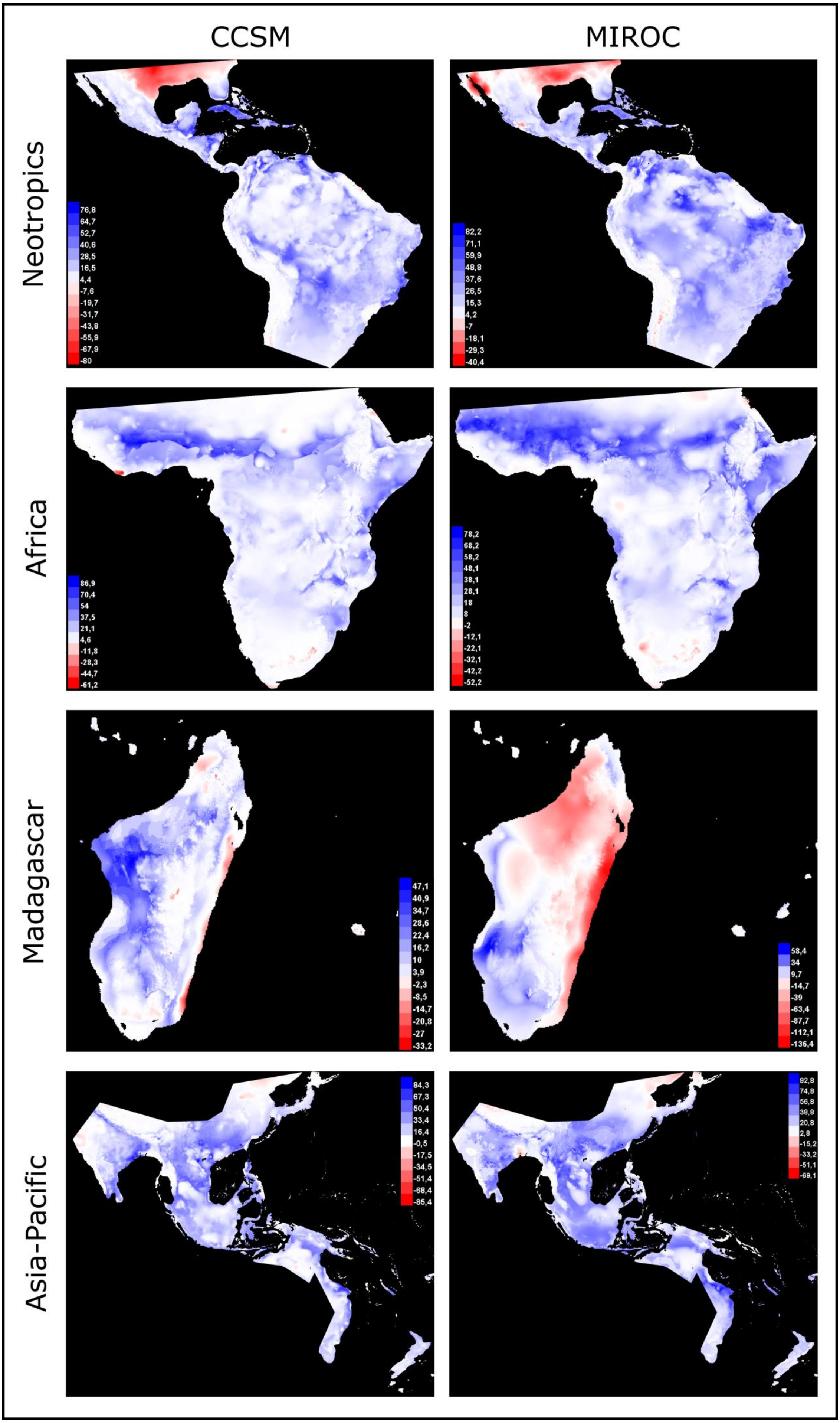
CCSM
MIROC
Neotropics
Africa
Madagascar
Asia-Pacific
76,8 64,7 52,7 40,6 28,5 16,5 4,4 -7,6 -19,7 -31,7 -43,8 -55,9 -67,9 -80
82,2 71,1 59,9 48,8 37,6 26,5 15,3 4,2 -7 -18,1 -29,3 -40,4
86,9 70,4 54 37,5 21,1 4,6 -11,8 -28,3 -44,7 -61,2
78,2 68,2 58,2 48,1 38,1 28,1 18 8 -2 -12,1 -22,1 -32,1 -42,2 -52,2
47,1 40,9 34,7 28,6 22,4 16,2 10 3,9 -2,3 -8,5 -14,7 -20,8 -27 -33,2
58,4 34 9,7 -14,7 -39 -63,4 -87,7 -112,1 -136,4
84,3 67,3 50,4 33,4 16,4 -0,5 -17,5 -34,5 -51,4 -68,4 -85,4
92,8 74,8 56,8 38,8 20,8 2,8 -15,2 -33,2 -51,1 -69,1

2

Figure Legends

Figure S1. All ages of the BEAST-derived species-level maximum clade credibility (MCC) chronogram based on ITS sequence data (see also Fig. 2), with branch lengths proportional to time in millions of years, including all 332 taxa (see Table S1 for list of all species included in the analysis). The estimated median age of each node is shown above branches and the range of the 95% HPD interval is indicated in brackets at nodes. Red circles at nodes indicate secondary calibration points (see text).

Figure S2. Posterior probabilities (PP) for the BEAST-derived species-level maximum clade credibility (MCC) chronogram (see Fig. 2).

Figure S3. LTT plots of 1,000 trees sampled from the posterior distribution of the BEAST analyses.

Figure S4. Current distribution models of the four *Bulbophyllum* lineages projected onto climatic conditions of the LGM derived from CCSM and MIROC, respectively. Predicted distribution probabilities are shown as logistic values of suitability above the region-specific maximum training sensitivity plus specificity (MTSS) thresholds. See also Figure 6B for the consensus prediction.

Figure S5. Representative Multivariate Environmental Similarity Surface (MESS) analyses of individual CCSM and MIROC models for LGM climatic conditions. Positive (blue) and negative (red) MESS scores indicate areas with and without current equivalents (i.e. analogue and no-analogue climates), respectively. Overall, these analyse showed relative good in agreement between the CCSM and MIROC climatic data (for the Neotropics, Africa, Asian-Pacific region and also to a lesser extent Madagascar) in terms of similarity between the presently observed climate, used to train the MAXENT model, and the LGM projected climate..

Supplementary Tables

Table S1. GenBank accession numbers and vouchers/references for 332 (nrDNA) ITS sequences of *Bulbophyllum* (320), *Dendrobium* (6) and *Epigeneium* (6), subdivided by geographic region. Note, this list includes 253 accessions obtained from GenBank (NCBI) plus 79 previously unpublished sequences of Gamisch *et al.* [48] (the latter marked in bold).

Species	GenBank number	Origin
Bulbophyllum alinae	**MH822366**	Africa
Bulbophyllum barbigerum	EF195918.1	Africa
Bulbophyllum bifarium	**MH822369**	Africa
Bulbophyllum calvum	**MH822373**	Africa
Bulbophyllum capituliflorum	**MH822375**	Africa
Bulbophyllum cochleatum	**MH822377**	Africa
Bulbophyllum cocoinum	**MH822378**	Africa
Bulbophyllum colubrinum	**MH822379**	Africa
Bulbophyllum comatum	**MH822380**	Africa
Bulbophyllum distans	**MH822383**	Africa
Bulbophyllum encephalodes	**MH822385**	Africa
Bulbophyllum falcatum	EF195927.1	Africa
Bulbophyllum fuscum	**MH822387**	Africa
Bulbophyllum hapalanthos	EF195980.1	Africa
Bulbophyllum imbricatum	**MH822388**	Africa
Bulbophyllum jaapii	**MH822389**	Africa
Bulbophyllum josephi	**MH822390**	Africa
Bulbophyllum kupense	**MH822392**	Africa
Bulbophyllum lizae	**MH822395**	Africa
Bulbophyllum lupulinum	EF195932.1	Africa
Bulbophyllum malawiense	KJ558759.1	Africa
Bulbophyllum maximum	**MH822399**	Africa
Bulbophyllum mayombeense	EF195934.1	Africa
Bulbophyllum mediocre	**MH822400**	Africa
Bulbophyllum nigritianum	**MH822405**	Africa
Bulbophyllum oreonastes	**MH822406**	Africa
Bulbophyllum oxychilum	EF195937.1	Africa
Bulbophyllum pipio	**MH822409**	Africa
Bulbophyllum porphyrostachys	**MH822410**	Africa
Bulbophyllum pumilum	**MH822413**	Africa
Bulbophyllum renkinianum	**MH822417**	Africa
Bulbophyllum sandersonii	**MH822418**	Africa

Bulbophyllum schimperianum	**MH822421**	Africa
Bulbophyllum schinzianum	**MH822422**	Africa
Bulbophyllum subligaculiferum	**MH822424**	Africa
Bulbophyllum teretifolium	**MH822425**	Africa
Bulbophyllum tetragonum	**MH822426**	Africa
Bulbophyllum thomense	**MH822428**	Africa
Bulbophyllum vanum	**MH822443**	Africa
Bulbophyllum scaberulum	**MH822419**	Africa, Asia
Bulbophyllum intertextum	EF195930.1	Africa, Madagascar
Adelopetalum bracteatum	AY239945.1	Asia
Bulbophyllum acuminatum	JF428117.1	Asia
Bulbophyllum affine	KC568305.1	Asia
Bulbophyllum alsiosum	EF195917.1	Asia
Bulbophyllum ambrosia	KC568306.1	Asia
Bulbophyllum amplifolium	KY022454.1	Asia
Bulbophyllum andersonii	JN619417.1	Asia
Bulbophyllum annandalei	KY966427.1	Asia
Bulbophyllum apodum	JF428121.1	Asia
Bulbophyllum armeniacum	JF428112.1	Asia
Bulbophyllum auratum	KY966428.1	Asia
Bulbophyllum auriculatum	**MH822430**	Asia
Bulbophyllum ayuthayense	KY966429.1	Asia
Bulbophyllum bicolor	KY966430.1	Asia
Bulbophyllum biflorum	EF195919.1	Asia
Bulbophyllum blepharistes	EU477512.1	Asia
Bulbophyllum bowkettae	**MH822370**	Asia
Bulbophyllum burfordiense	AY273716.1	Asia
Bulbophyllum cambodianum	KM924469.1	Asia
Bulbophyllum cameronense	**MH822431**	Asia
Bulbophyllum careyanum	KY966435.1	Asia
Bulbophyllum cariniflorum	KF866243.1	Asia
Bulbophyllum caudatisepalum	JF428108.1	Asia
Bulbophyllum cheiropetalum	JF428109.1	Asia
Bulbophyllum clandestinum	KY966436.1	Asia
Bulbophyllum claptonense	**MH822432**	Asia
Bulbophyllum colomaculosum	JN619412.1	Asia
Bulbophyllum concinnum	KY966437.1	Asia
Bulbophyllum congestum	KY966438.1	Asia
Bulbophyllum coweniorum	**MH822433**	Asia
Bulbophyllum cumingii	EF195923.1	Asia

Bulbophyllum cylindraceum	KX455816.1	Asia
Bulbophyllum dayanum	JF428119.1	Asia
Bulbophyllum dearei	EF195924.1	Asia
Bulbophyllum delitescens	KY966440.1	Asia
Bulbophyllum disciflorum	KY966443.1	Asia
Bulbophyllum drymoglossum	KT338651.1	Asia
Bulbophyllum emiliorum	EF195926.1	Asia
Bulbophyllum exiguum	**MH822386**	Asia
Bulbophyllum facetum	**MH822434**	Asia
Bulbophyllum flavescens	JF428102.1	Asia
Bulbophyllum frostii	KY966445.1	Asia
Bulbophyllum gracillimum	KY966446.1	Asia
Bulbophyllum guttulatum	KF866245.1	Asia
Bulbophyllum hamatipes	EF195929.1	Asia
Bulbophyllum haniffii	KY966447.1	Asia
Bulbophyllum helenae	KF866240.1	Asia
Bulbophyllum hiepii	**MH822435**	Asia
Bulbophyllum hirtulum	JF428126.1	Asia
Bulbophyllum hirtum	KY966448.1	Asia
Bulbophyllum hirundinis	KY022456.1	Asia
Bulbophyllum hyalosemoides	**MH822436**	Asia
Bulbophyllum inconspicuum	AB786895.1	Asia
Bulbophyllum inunctum	JF428110.1	Asia
Bulbophyllum japonicum	AB786894.1	Asia
Bulbophyllum kwangtungense	JN619414.1	Asia
Bulbophyllum lasianthum	JF428116.1	Asia
Bulbophyllum laxiflorum	KY966449.1	Asia
Bulbophyllum leion	KM924484.1	Asia
Bulbophyllum lemniscatoides	KY966450.1	Asia
Bulbophyllum leopardinum	**MH822394**	Asia
Bulbophyllum lepidum	KY966452.1	Asia
Bulbophyllum levinei	JN619409.1	Asia
Bulbophyllum lilacinum	JF428111.1	Asia
Bulbophyllum limbatum	JF428122.1	Asia
Bulbophyllum lindleyanum	KY966454.1	Asia
Bulbophyllum linearifolium	JF428103.1	Asia
Bulbophyllum lipingtaoi	KX455817.1	Asia
Bulbophyllum lobbii	EF195931.1	Asia
Bulbophyllum longibrachiatum	KY022457.1	Asia
Bulbophyllum longissimum	KY966458.1	Asia

Bulbophyllum lopalanthum	KY966459.1	Asia
Bulbophyllum macphersonii	**MH822397**	Asia
Bulbophyllum macranthum	EF195933.1	Asia
Bulbophyllum mastersianum	KY966460.1	Asia
Bulbophyllum medusae	KY966461.1	Asia
Bulbophyllum melanoglossum	KY022458.1	Asia
Bulbophyllum membranacum	**MH822401**	Asia
Bulbophyllum membranifolium	EF195935.1	Asia
Bulbophyllum meson	KM924490.1	Asia
Bulbophyllum microglossum	**MH822437**	Asia
Bulbophyllum minutissimum	**MH822402**	Asia
Bulbophyllum monanthos	KY966462.1	Asia
Bulbophyllum monanthum	**MH822438**	Asia
Bulbophyllum mutabile	JF428101.1	Asia
Bulbophyllum nasutum	KM924486.1	Asia
Bulbophyllum newportii	JF706720.1	Asia
Bulbophyllum nigrescens	KX455818.1	Asia
Bulbophyllum nodosum	KF866241.1	Asia
Bulbophyllum nummularia	KY966464.1	Asia
Bulbophyllum oblongum	KM924473.1	Asia
Bulbophyllum obtusangulum	JN619410.1	Asia
Bulbophyllum odoratum	JF428113.1	Asia
Bulbophyllum omerandrum	JN619419.1	Asia
Bulbophyllum orectopetalum	EF195936.1	Asia
Bulbophyllum orientale	KY966468.1	Asia
Bulbophyllum ovalifolium	JF428104.1	Asia
Bulbophyllum palawanense	**MH822439**	Asia
Bulbophyllum patens	EF195938.1	Asia
Bulbophyllum pecten-veneris	KY966471.1	Asia
Bulbophyllum pictum	KY966472.1	Asia
Bulbophyllum picturatum	EF195939.1	Asia
Bulbophyllum piestoglossum	**MH822440**	Asia
Bulbophyllum pileatum	JF428105.1	Asia
Bulbophyllum pingtungense	KY022459.1	Asia
Bulbophyllum polystictum	**MH822441**	Asia
Bulbophyllum purpurascens	KY966473.1	Asia
Bulbophyllum radicans	**MH822416**	Asia
Bulbophyllum repens	KY966475.1	Asia
Bulbophyllum reptans	JN114443.1	Asia
Bulbophyllum restrepia	JF428123.1	Asia

Bulbophyllum roseum	KM924485.1	Asia
Bulbophyllum rothschildianum	KX455819.1	Asia
Bulbophyllum sanguineopunctatum	KY966476.1	Asia
Bulbophyllum schillerianum	**MH822420**	Asia
Bulbophyllum siamense	EF195942.1	Asia
Bulbophyllum smitinandii	EF195943.1	Asia
Bulbophyllum sp.	**MH822414**	Asia
Bulbophyllum spathulatum	KY966478.1	Asia
Bulbophyllum sukhakulii	KY966481.1	Asia
Bulbophyllum sulcatum	JF428106.1	Asia
Bulbophyllum sumatranum	**MH822442**	Asia
Bulbophyllum sutepense	KY966482.1	Asia
Bulbophyllum taiwanense	KY022460.1	Asia
Bulbophyllum tianguii	JN619415.1	Asia
Bulbophyllum tigridum	KX455820.1	Asia
Bulbophyllum triste	KY966483.1	Asia
Bulbophyllum umbellatum	KY966484.1	Asia
Bulbophyllum uniflorum	JF428098.1	Asia
Bulbophyllum wallichii	JN619411.1	Asia
Bulbophyllum wendlandianum	KY966485.1	Asia
Bulbophyllum yunnanense	KX455822.1	Asia
Bulbophyllum longiflorum	EF196023.1	Asia, Madagascar, Africa
Oxysepala ovalifolia	AY240018.1	Asia
Bulbophyllum aff. baronii	EF195947.1	Madagascar
Bulbophyllum aff. sphaerobulbum	EF195950.1	Madagascar
Bulbophyllum afzelii	**MH822365**	Madagascar
Bulbophyllum aggregatum	EF195951.1	Madagascar
Bulbophyllum alexandrae	EF195952.1	Madagascar
Bulbophyllum alleizettei	EF195953.1	Madagascar
Bulbophyllum ambatoavense	EF195954.1	Madagascar
Bulbophyllum amoenum	EF195955.1	Madagascar
Bulbophyllum amparihibense	**MH822367**	Madagascar
Bulbophyllum analamazoatrae	EF195957.1	Madagascar
Bulbophyllum anjozorobeense	EF195958.1	Madagascar
Bulbophyllum ankaizinense	EF195959.1	Madagascar
Bulbophyllum approximatum	EF195960.1	Madagascar
Bulbophyllum aubrevillei	EF195962.1	Madagascar
Bulbophyllum auriflorum	EF195963.1	Madagascar

Bulbophyllum bathieanum	**MH822368**	Madagascar
Bulbophyllum bicoloratum	EF195964.1	Madagascar
Bulbophyllum bryophytoides	**MH822371**	Madagascar
Bulbophyllum callosum	**MH822372**	Madagascar
Bulbophyllum calyptratum	**MH822374**	Madagascar
Bulbophyllum calyptropus	EF195965.1	Madagascar
Bulbophyllum capuronii	EF195966.1	Madagascar
Bulbophyllum cardiobulbum	KJ558721.1	Madagascar
Bulbophyllum ciliatilabrum	EF195968.1	Madagascar
Bulbophyllum cirrhoglossum	KJ558728.1	Madagascar
Bulbophyllum coccinatum	**MH822376**	Madagascar
Bulbophyllum complanatum	KJ558750.1	Madagascar
Bulbophyllum conchidioides	EF195969.1	Madagascar
Bulbophyllum coriophorum	EF195970.1	Madagascar
Bulbophyllum cyclanthum	EF195971.1	Madagascar
Bulbophyllum debile	**MH822381**	Madagascar
Bulbophyllum densum	**MH822382**	Madagascar
Bulbophyllum divaricatum	**MH822384**	Madagascar
Bulbophyllum edentatum	EF195972.1	Madagascar
Bulbophyllum erectum	KJ558716.1	Madagascar
Bulbophyllum francoisii	EF195977.1	Madagascar
Bulbophyllum hamelinii	EF195979.1	Madagascar
Bulbophyllum henrici	EF195981.1	Madagascar
Bulbophyllum hildebrandtii	KJ558720.1	Madagascar
Bulbophyllum histrionicum	KJ558758.1	Madagascar
Bulbophyllum horizontale	KJ558712.1	Madagascar
Bulbophyllum humbertii	EF195985.1	Madagascar
Bulbophyllum imerinense	EF195987.1	Madagascar
Bulbophyllum implexum	KJ558757.1	Madagascar
Bulbophyllum incurvum	KJ558718.1	Madagascar
Bulbophyllum insolitum	EF196019.1	Madagascar
Bulbophyllum jumelleanum	**MH822391**	Madagascar
Bulbophyllum lakatoense	**MH822393**	Madagascar
Bulbophyllum leandrianum	EF196021.1	Madagascar
Bulbophyllum lecouflei	KJ558723.1	Madagascar
Bulbophyllum liparidioides	EF196022.1	Madagascar
Bulbophyllum longivaginans	EF196025.1	Madagascar
Bulbophyllum luteobracteatum	KJ558738.1	Madagascar
Bulbophyllum lyperocephalum	**MH822396**	Madagascar
Bulbophyllum mangenotii	**MH822398**	Madagascar

Bulbophyllum marovoense	EF196027.1	Madagascar
Bulbophyllum melleum	EF196030.1	Madagascar
Bulbophyllum minutum	EF196031.1	Madagascar
Bulbophyllum molossus	EF196032.1	Madagascar
Bulbophyllum moratii	**MH822403**	Madagascar
Bulbophyllum muscicola	EF196033.1	Madagascar
Bulbophyllum namoronae	**MH822404**	Madagascar
Bulbophyllum nitens	EF196034.1	Madagascar
Bulbophyllum nutans	EF196036.1	Madagascar
Bulbophyllum obtusatum	EF196039.1	Madagascar
Bulbophyllum occlusum	EF196040.1	Madagascar
Bulbophyllum occultum	KJ558760.1	Madagascar
Bulbophyllum ochrochlamys	EF196042.1	Madagascar
Bulbophyllum oreodorum	EF196043.1	Madagascar
Bulbophyllum ormerodianum	**MH822407**	Madagascar
Bulbophyllum oxycalyx	EF196044.1	Madagascar
Bulbophyllum pachypus	EF196047.1	Madagascar
Bulbophyllum paleiferum	**MH822408**	Madagascar
Bulbophyllum pantoblepharon	EF196048.1	Madagascar
Bulbophyllum pervillei	EF196049.1	Madagascar
Bulbophyllum peyrotii	EF196051.1	Madagascar
Bulbophyllum platypodum	EF196052.1	Madagascar
Bulbophyllum pleurothallopsis	EF196053.1	Madagascar
Bulbophyllum prismaticum	**MH822411**	Madagascar
Bulbophyllum protectum	**MH822412**	Madagascar
Bulbophyllum quadrialatum	**MH822415**	Madagascar
Bulbophyllum quadrifarium	KJ558754.1	Madagascar
Bulbophyllum rauhii	EF196055.1	Madagascar
Bulbophyllum rubiginosum	EF196056.1	Madagascar
Bulbophyllum rubrum	KJ558741.1	Madagascar
Bulbophyllum ruginosum	KJ558751.1	Madagascar
Bulbophyllum sambiranense	KJ558746.1	Madagascar
Bulbophyllum sandrangatense	EF196058.1	Madagascar
Bulbophyllum sciaphile	EF196059.1	Madagascar
Bulbophyllum senghasii	KJ558727.1	Madagascar
Bulbophyllum subclavatum	**MH822423**	Madagascar
Bulbophyllum sulfureum	EF196071.1	Madagascar
Bulbophyllum therezienii	**MH822427**	Madagascar
Bulbophyllum trifarium	KJ558719.1	Madagascar
Bulbophyllum turkii	EF196073.1	Madagascar

Bulbophyllum variegatum	KJ558713.1	Madagascar
Bulbophyllum variifolium	**MH822429**	Madagascar
Bulbophyllum ventriosum	EF196075.1	Madagascar
Bulbophyllum vestitum	EF196076.1	Madagascar
Bulbophyllum elliottii	EF195976.1	Madagascar, Africa
Bulbophyllum pusillum	KJ558730.1	Madagascar, Africa
Bulbophyllum humblotii	EF195986.1	Madagascar, Africa
Bulbophyllum adiamantinum	GQ339691.1	Neotropics
Bulbophyllum aff. atropurpureum	GQ862980.1	Neotropics
Bulbophyllum aff. chloropterum	EF195921.1	Neotropics
Bulbophyllum atropurpureum	GQ339706.1	Neotropics
Bulbophyllum bidentatum	GQ339701.1	Neotropics
Bulbophyllum boudetianum	GQ339723.1	Neotropics
Bulbophyllum bracteolatum	EF195920.1	Neotropics
Bulbophyllum campos-portoi	GQ339721.1	Neotropics
Bulbophyllum cantagallense	GQ339722.1	Neotropics
Bulbophyllum carassense	GQ339717.1	Neotropics
Bulbophyllum chloroglossum	GQ339694.1	Neotropics
Bulbophyllum cilulia	GQ339698.1	Neotropics
Bulbophyllum cirrhosum	GQ339685.1	Neotropics
Bulbophyllum cribbianum	EF195922.1	Neotropics
Bulbophyllum epiphytum	GQ339693.1	Neotropics
Bulbophyllum exaltatum	GQ339715.1	Neotropics
Bulbophyllum filifolium	GQ339699.1	Neotropics
Bulbophyllum gladiatum	GQ339718.1	Neotropics
Bulbophyllum glutinosum	EF195928.1	Neotropics
Bulbophyllum hoehnei	GQ339700.1	Neotropics
Bulbophyllum insectiferum	GQ339692.1	Neotropics
Bulbophyllum kautskyi	GQ339705.1	Neotropics
Bulbophyllum malachadenia	GQ339708.1	Neotropics
Bulbophyllum manarae	GQ339704.1	Neotropics
Bulbophyllum melloi	GQ339719.1	Neotropics
Bulbophyllum mentosum	GQ339690.1	Neotropics
Bulbophyllum meridense	GQ339712.1	Neotropics
Bulbophyllum micranthum	GQ339697.1	Neotropics
Bulbophyllum micropetaliforme	GQ339709.1	Neotropics
Bulbophyllum mucronifolium	GQ339695.1	Neotropics
Bulbophyllum nagelii	GQ339720.1	Neotropics
Bulbophyllum napellii	GQ339711.1	Neotropics

Bulbophyllum perii	GQ862815.1	Neotropics
Bulbophyllum pitengoense	KC581944.1	Neotropics
Bulbophyllum plumosum	EF195941.1	Neotropics
Bulbophyllum regnellii	GQ339710.1	Neotropics
Bulbophyllum rupicola	GQ339696.1	Neotropics
Bulbophyllum setigerum	GQ339689.1	Neotropics
Bulbophyllum steyermarkii	GQ339688.1	Neotropics
Bulbophyllum teimosense	GQ339703.1	Neotropics
Bulbophyllum tripetalum	GQ339716.1	Neotropics
Bulbophyllum uhl-gabrielianum	KC581945.1	Neotropics
Bulbophyllum weddellii	GQ339713.1	Neotropics
Dendrobium brymerianum	KF143432.1	Asia
Dendrobium exile	KF143456.1	Asia
Dendrobium lancifolium	AY239976.1	Asia
Dendrobium stockeri	AB593667.1	Asia
Dendrobium trigonopus	KF143521.1	Asia
Winika cunninghamii	AY240019.1	Asia
Epigeneium amplum	AY240010.1	Asia
Epigeneium clemensiae	KF143530.1	Asia
Epigeneium cymbidioides	AY240011.1	Asia
Epigeneium nakaharaei	AF521618.1	Asia
Epigeneium tricallosum	JF706721.1	Asia
Epigeneium triflorum	AY240013.1	Asia

Table S2. Statistical support for ancestral areas against alternative states of three key nodes (i.e. I: the crown node of *Bulbophyllum*, II: the stem node of the Madagascan, African and Neotropical lineages, and III: the stem node of the African and Neotropical lineages; see also Fig. 2) using 2 logarithmic Bayes Factor (BF) comparisons. Positive values indicate support of the rows model compared to the columns model. A: Asia-Pacific; B: Madagascar; C: Neotropics; D: Africa.

	**Marginal likelihood**	**S. E.**		**2 (log BF)**		
**Restricted node**			I restricted to A	I restricted to B	I restricted to C	I restricted to D
I restricted to A	-28.603	+/- 0.033	-	**11.28**	**15.63**	**11.566**
I restricted to B	-34.24	+/- 0.047	-11.276	-	4.352	0.29
I restricted to C	-36.416	+/- 0.161	-15.628	-4.352	-	-4.062
I restricted to D	-34.386	+/- 0.099	-11.566	-0.29	4.062	-
			II restricted to A	II restricted to B	II restricted to C	II restricted to D
II restricted to A	-30.106	+/- 0.022	-	-1.372	-0.102	0.41
II restricted to B	-29.42	+/- 0.096	**1.372**	-	**1.27**	**1.782**
II restricted to C	-30.055	+/- 0.028	0.102	-1.27	-	0.512
II restricted to D	-30.311	+/- 0.103	-0.41	-1.782	-0.512	-
			III restricted to A	III restricted to B	III restricted to C	III restricted to D
III restricted to A	-33.827	+/- 0.123	-	-6.742	-8.272	-8.076
III restricted to B	-30.456	+/- 0.078	**6.742**	-	-1.528	-1.332
III restricted to C	-29.692	+/- 0.071	**8.272**	**1.528**	-	**0.196**
III restricted to D	-29.79	+/- 0.07	**8.076**	**1.332**	-0.196	-

Table S3. Three biogeographic models compared by BioGeoBEARS as implemented in RASP. Models are ranked from best to worst, according to dAICc scores (best model marked in bold). See Table S4 for abbreviations.

**Model**	**Nb**	**LH**	**AICc**	**dAICc**
**DEC**	**2**	**-69.4**	**142,8**	**0**
DIVALIKE	2	-71.33	146,7	3,9
BAYAREALIKE	2	-98.69	201,4	58,6

Table S4. Nine diversification models fitted to the crown groups of the Madagascan, African and Neotropical *Bulbophyllum* lineages (see Fig. 2), using RPANDA v. 1.3. Models are ranked from best to worst, according to AICc scores. Note that a constant rate speciation and extinction (birth-death) model (marked bold) was favoured in the case of Madagascar and the Neotropics, and performed not significantly worse than the best-fitting model for Africa (no extinction and constant speciation rate). For each clade (lineage), sample frequencies are shown in parentheses as number of sampled/extant species (the latter according to Sieder *et al*. 2009).

**Madagascar (103/210)**				
**Description**	**Nb**	**LH**	**AICc**	**dAICc**
**Constant speciation and extinction rates**	**2**	**-188.4508**	**381.0217**	**0**
No extinction and exponential variation in speciation rate through time	2	-188.6469	381.4138	0.39
Exponential variation in speciation and constant extinction rates	4	-188.1023	382.447	1.43
Constant speciation rate and linear variation in extinction rate	3	-188.1203	382.4831	1.46
Linear variation in speciation rate and constant extinction rate	3	-188.1206	382.4836	1.46
Constant speciation rate and exponential variation in extinction rate	3	-188.1304	382.5033	1.48
No extinction and linear variation in speciation rate	2	-190.1134	384.3468	3.33
Exponential variation in speciation and extinction rates	4	-188.0805	384.5693	3.55
No extinction and constant speciation rate	1	-196.6185	395.2766	14.25
**Neotropic (43/94)**				
**Description**	**Nb**	**LH**	**AICc**	**dAICc**
**Constant speciation and extinction rates**	**2**	**-82.21047**	**168.7209**	**0**
Exponential variation in speciation and constant extinction rates	4	-81.82851	170.2724	1.55
Constant speciation rate and linear variation in extinction rate	3	-81.82963	170.2746	1.55
Linear variation in speciation rate and constant extinction rate	3	-81.8297	170.2748	1.55
Constant speciation rate and exponential variation in extinction rate	3	-81.83751	170.2904	1.57
No extinction and exponential variation in speciation rate through time	2	-84.05735	172.4147	3.69
Exponential variation in speciation and extinction rates	4	-81.99867	173.05	4.33
No extinction and linear variation in speciation rate	2	-85.30366	174.9073	6.19
No extinction and constant speciation rate	1	-88.53768	179.1729	10.45
**Africa (40/80)**				
**Description**	**Nb**	**LH**	**AICc**	**dAICc**
No extinction and constant speciation rate	1	-77.5409	157.19	0
No extinction and linear variation in speciation rate	2	-76.60034	157.53	0.34
No extinction and exponential variation in speciation rate through time	2	-76.64988	157.62	0.44
**Constant speciation and extinction rates**	**2**	**-76.82769**	**157.98**	**0.79**
Constant speciation rate and exponential variation in extinction rate	3	-76.53411	159.73	2.55
Linear variation in speciation rate and constant extinction rate	3	-76.60036	159.87	2.68
Constant speciation rate and linear variation in extinction rate	3	-76.61756	159.90	2.71
Exponential variation in speciation and constant extinction rates	4	-76.64964	159.97	2.78
Exponential variation in speciation and extinction rates	4	-76.64988	162.44	5.26

Abbreviations: Nb, number of parameters per model; LH, log-likelihood; AICc, Akaike's Information Criterion corrected for finite sample size; ΔAICc, difference in the AICc scores between the alternative model and the model with the lowest AICc.

Table S5. Bioclimatic variables used for the ecological niche modelling (ENM) of *Bulbophyllum* lineages per region and after excluding highly redundant variables (Pearson's correlation > 0.9).

Bioclimatic variable	Neotropics	Africa	Madagascar	Asia-Pacific
BIO1 = Annual mean temperature	x	x	x	x
BIO3 = Isothermality	x			
BIO5 = Max. temperature of warmest month	x	x	x	x
BIO6 = Min. temperature of coldest month	x	x	x	x
BIO7 = Temperature annual range	x	x	x	x
BIO8 = Mean temperature of wettest quarter		x	x	x
BIO9 = Mean temperature of driest quarter	x	x	x	x
BIO11 = Mean temperature of coldest quarter			x	
BIO12 = Annual precipitation	x	x		
BIO13 = Precipitation of wettest month	x	x	x	x
BIO14 = Precipitation of driest month	x	x	x	x
BIO15 = Precipitation seasonality			x	
BIO19 = Precipitation of coldest quarter			x	
